# Bio‐Inspired Stored Magnetic Energy Actuator with Transient Triggering and Programmable Logic

**DOI:** 10.1002/advs.202509683

**Published:** 2025-08-04

**Authors:** Chao Xu, Shuo Jiang, Lu Zhang, Xueli Zhou, Qingping Liu, Luquan Ren

**Affiliations:** ^1^ Key Laboratory of Bionic Engineering Jilin University Changchun 130025 China; ^2^ Liaoning Academy of Materials Shenyang 110167 China; ^3^ Weihai Institute for Bionics Jilin University Weihai 264207 China; ^4^ College of Construction Engineering Jilin University Changchun 130026 China

**Keywords:** bio‐inspired actuators, directed magnetization techniques, environmental logic gating, magnetically mediated energy storage, transient triggering

## Abstract

Smart actuators generally face the problems of limited response speed, dependence on external energy supply, and lack of multi‐environmental signal logic judgment capability. Therefore, this study proposes a new paradigm of bionic magnetic energy actuator, which realizes high‐speed autonomous response without external energy source by fusing magnetic transient triggering with programmable environmental logic gating strategy. Based on direct ink writing 3D printing technology, an integrated structure of “magnetic energy storage‐environmental unlocking” is constructed by combining the directional magnetization design of a hard magnetic material (NdFeB/polydimethylsiloxane) with an environment‐responsive locking material (phase‐change wax, polyvinylpyrrolidone/ethanol solution). This structure synergizes the slow response of environmental signals with the transient release of magnetic energy at millisecond level, and the release rate of magnetic energy is several orders of magnitude higher than that of the traditional stimulus‐responsive actuator; meanwhile, based on the biomimetic “and gate” logic coding, the precise conditional judgment of time‐sequential environmental signals (e.g., “high temperature → rain”) has been realized. The results show that the actuator can simulate high‐speed seed ejection, multimodal gripping, and logic‐gated bouncing behaviors of biological prototypes. This research provides new ideas for environmentally adaptive robots in wild scenarios, with potential applications in ecological restoration and precision agriculture.

## Graphical abstract

A bio‐inspired magnetic energy actuator achieves 1.6 m/s transient release—three orders faster than conventional systems—via direct ink writing printed NdFeB/Polydimethylsiloxane composites and environmental locks; biomimetic AND‐gate logic enables sequential cue recognition (e.g., “temperature → rainfall”). Optimized magnetic inks exhibit critical shear‐thinning, while cuboid energy units deliver 619 mN force and 1.84 mN·m torque for robust self‐alignment.

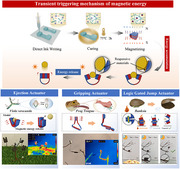

## 1 Introduction

Over billions of years of evolution, natural organisms have developed sophisticated environmental adaptation strategies. These natural systems offer valuable design principles for artificial actuators, where rapid actuation is crucial. While storing energy for a long period of time, millisecond‐level action responses are realized through transient triggering by natural conditions.^[^ ^1^, ^2^, ^3^ ^]^ For example, witch hazel seed pods shrink and split during drying, and the inner wall squeezes the seeds, allowing them to be ejected several meters away with a maximum velocity of 12.3 m s^−1^.^[^ ^4^ ^]^ Mantis shrimp store energy through muscle‐joint synergies and release instantaneous kinetic energy during hunting, with velocities of up to 23 m s^−1^.^[^ ^5^ ^]^ The core of these systems lies in the precise coupling of the energy storage medium to the triggering conditions. Through structural evolution, organisms store energy stably in fibers, muscles, and other media for long periods of time, and trigger the release of energy by transient changes in natural signals such as temperature and humidity. This “energy storage‐trigger” integration mechanism provides an idea for the design of bionic actuators, but it also poses a challenge to the material design, energy control and environmental response of human industrial systems.

Bionic actuators realize intelligent actuation by mimicking the movement mechanism of living organisms,^[^ ^6^, ^7^, ^8^, ^9^, ^10^, ^11^ ^]^ combined with stimulus‐responsive materials.^[^ ^12^, ^13^, ^14^, ^15^, ^16^, ^17^ ^]^ In particular, rapid advances in 3D printing have enabled the fabrication of anisotropic and site‐specific materials, greatly increasing the capabilities of bionic actuators.^[^ ^18^, ^19^, ^20^ ^]^ For example, Jennifer A. Lewis et al. mimic the oriented arrangement of cellulose in plant cell walls using a shear‐induced fiber orientation process with direct ink writing (DIW). The prepared smart actuator spontaneously deforms into a complex three‐dimensional form upon water immersion.^[^ ^21^ ^]^ Further, the team developed a rotational multi‐material 3D printing techniques that allow voxel‐level control of material distribution, enabling the fabrication of helical dielectric actuators capable of axial shrinkage and torsion under electric fields.^[^ ^22^ ^]^ Hang Jerry Qi et al. precisely regulated the spatial cross‐linking density and glass transition temperature of shape memory polymers through digital light processing, combined with pneumatic loading, to achieve controlled deformation of complex 3D structures.^[^ ^23^ ^]^ Jiayu Liu et al. developed a bionic dual‐gel 4D printing technique to realize the precise construction of heterogeneous materials by alternating thermo‐responsive pNIPAM and inert pAAM gels. The resulting tubular actuator exhibits multimodal deformation under temperature control, precisely reproducing the grasping action of coral tentacles.^[^ ^24^ ^]^ 3D printed bionic actuators have potential for wide applications in fields such as soft robotics,^[^ ^25^, ^26^, ^27^, ^28^, ^29^, ^30^, ^31^ ^]^ biomedical engineering.^[^ ^32^, ^33^, ^34^, ^35^, ^36^, ^37^ ^]^

**Figure 1:**
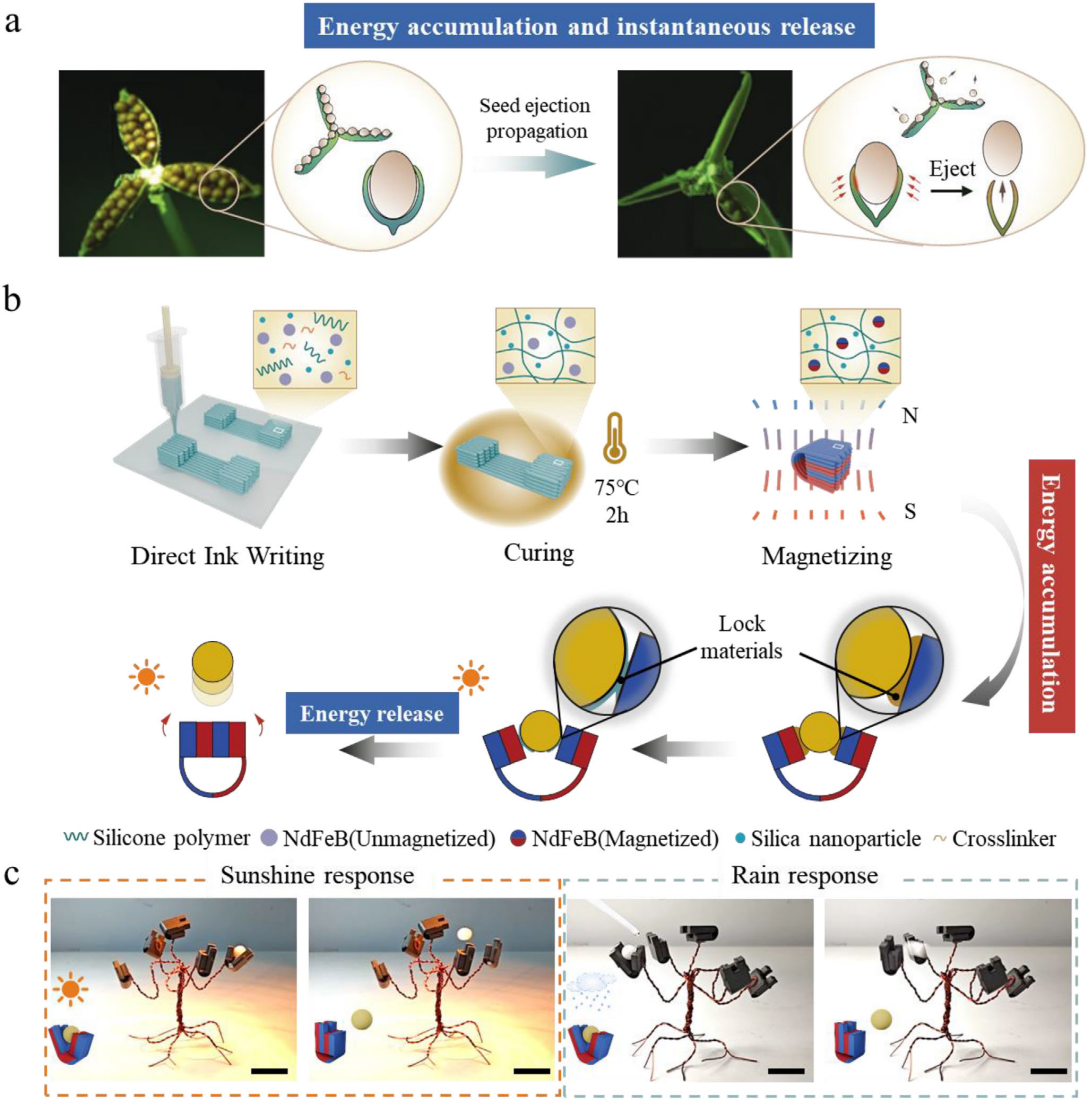
Design principle of the Bio‐inspired stored magnetic energy actuator with natural condition response mechanism. a) Schematic diagram of the whole process of *Viola verecunda* seed pods from seed encapsulation (growth period) to mature ejection (energy storage release period).^[^ ^44^ ^]^ b) Schematic diagram of the fabrication process and triggering of the DIW‐based stored magnetic energy actuator (blue: N‐pole, red: S‐pole). c) High temperature triggering mechanism: the yellow light simulated sunlight (45 °C) causing failure of the thermo‐responsive material and thus seed ejection (total response time: 85 s). And humidity trigger mechanism: glue tip burette simulating rainfall dissolved water response material triggering seed ejection (total response time: 135 s, scale: 25 mm).

Despite significant progress in bionic actuator research, existing technologies still face three major bottlenecks. First, the energy release rate is limited by the intrinsic kinetics of the material. Typical stimulus‐responsive materials (e.g., humidity‐driven hydrogels, thermo‐responsive liquid crystal elastomers) are limited by their intrinsic phase transition or diffusion dynamics, and the response time is usually in the order of seconds or even minutes, resulting in a limited deformation rate. In contrast, biological systems adopt the strategy of “slow response for energy storage, fast release for execution”, which breaks through the limitations of the intrinsic dynamics of materials and realizes the speed beyond the physiological limit (**Figure** **1**a). Second, energy release is highly dependent on external energy supply. Existing systems need to be triggered by high‐power lasers, light sources of specific wavelengths, or precision magnetic field control^[^ ^38^, ^39^, ^40^, ^41^ ^]^ (Figure 1b), and this active energy supply mode limits the application in infrastructure‐less scenarios such as the wilderness and disaster sites. Third, current systems lack the capacity for synergistic multi‐stimuli responses. Biological systems can be precisely regulated by sequential changes in temperature and humidity (e.g., the seed pods of *Banksia ericifolia* need to be triggered by the sequence of “forest fire‐rainfall”), while artificial systems are mostly limited to the passive response to a single stimulus,^[^ ^11^, ^12^, ^42^, ^43^ ^]^ and lack of the ability to logically judge the composite environmental signals, which severely limits the practical deployment of bionic actuators in complex real‐world environments such as ecological restoration or precision agriculture.

Aiming at the above challenges, this study proposes an innovative path of synergy between stored magnetic energy and 3D printing to realize the integrated design of bionic actuator with passive, fast response and logic judgment. Through three key breakthroughs: 1) magnetic energy‐mediated transient triggering mechanism: a hard magnetic material (NdFeB) is used to construct a heteromorphic magnetized structure, which utilizes magnetic pole attraction/repulsion to achieve a transient energy release of 1.6 m s^−1^, and the response speed is improved by three orders of magnitude compared with traditional materials. 2) Long‐term energy storage driven by environmental locking clasps: based on the DIW 3D printing technology, the integration of phase change wax (temperature response) and polyvinylpyrrolidone (PVP) ethanol solution (humidity response), design “stored magnetic energy‐environmental unlocking” composite structure, in the absence of external energy supply to achieve >6 months of stable energy storage. 3) Bionic logic gated multi‐conditional response: the development of the composite system of calcium carbide‐PVP‐paraffin wax, to build “and gate” locking structure, so that the actuator is only activated under specific sequential environmental signals such as “high temperature→ rainfall”, and remains silent when there is no critical condition or wrong sequence. Experiments show that the actuator can simulate the transient ejection of *Viola verecunda* seed pods, the multimodal gripping of frog tongues, and the logic‐responsive seeding of *Banksia ericifolia*, which provides a new paradigm for environment‐adaptive robots.

## 2 Results and Discussion

### 2.1 Transient Triggering Strategy for Magnetically Mediated Soft Actuators

When exploring the instantaneous triggering strategy of magnetic energy‐mediated soft actuator, we select the imitation *Viola verecunda* soft actuator as a typical case to analyze in order to show its working principle and characteristics comprehensively. *Viola verecunda*, a perennial herb, is conspicuously characterized by having three flattened elongated seed pods as shown in Figure 1a.^[^ ^44^ ^]^ The seed ejection mechanism of *Viola verecunda* seed pods is unique, relying on a moisture‐sensitive fiber structure for energy storage and rapid release. During seed pod maturation, the internal fibers possess the ability to contract directionally due to their anisotropic arrangement, and gradually accumulate elastic potential energy during dehydration. When the force on the seed reaches the critical threshold of homeostasis after maturation, the seed pod undergoes mechanical instability, and the seed is rapidly ejected at a speed of 12.3 m s^−1^, while the pod simultaneously contracts to release the stored energy. This mechanism combines the long‐term energy storage stability of the moisture‐sensitive structure of the fiber with the transient triggered responsiveness of the mechanical phase transition at the critical moisture threshold to achieve millisecond‐level fast motion, providing a natural example of passive energy control for bionic actuators.

Inspired by this, this study used DIW technology to prepare stored magnetic energy actuator, with polydimethylsiloxane (PDMS) as the flexible matrix. The ink's shear‐thinning behavior was tuned by incorporating unmagnetized neodymium‐iron‐boron (NdFeB) particles and fumed silica. After printing, the structure is cured at 75 °C for 2 h to form a crosslinked network structure and fix the distribution of magnetic particles. Subsequently, the shape of the printed structure is altered by external force, and the structure is magnetized in situ by placing it in a 2.5 T directional magnetic field to achieve the regular arrangement of N‐pole (blue) and S‐pole (red). The actuator critical bonding site integrates phase change wax (temperature responsive) or PVP/ethanol solution (humidity responsive) as the environmental locking material, as shown in Figure S1 (Supporting Information).

The actuator stores energy through attractive or repulsive forces between the magnetic poles, and failure of the environmental locking material (e.g., phase change wax or PVP/ethanol solution) at a specific temperature (above 37 °C, Figure 1b) or humidity threshold (simulated rainfall) triggers a transient release of magnetic energy. When the external environment reaches the threshold, the locking material melts or dissolves, the magnetic poles on both sides of the actuator close rapidly within milliseconds, and the seeds are ejected at high speed. Specifically, Figure 1c presents the temperature response process of a stored magnetic energy actuator. The process is realized with the help of integrating phase change wax as the locking material. When the ambient temperature gradually increases to 37 °C using a yellow light to simulate sunlight, the paraffin wax starts to melt, the fixation between the seed and the actuator is weakening, the seed slides slowly inside, and the attraction between the magnetic poles (N‐pole and S‐pole) of the actuator is gradually increasing. When the paraffin wax continues to melt and the seed moves to a specific position, the locking material reaches the failure threshold, at which time the magnetic energy is instantly released, the two sides of the magnetic poles are rapidly closed, and resulting in a rapid, high‐speed ejection of the seed within milliseconds. It is worth noting that response time covers the entire process from the environmental trigger signal to the failure of the locking material; transient time refers specifically to the duration of the magnetic energy release phase.

Next, Figure 1d illustrates the water response mechanism of a soft actuator. The water response of this actuator relies on a PVP / ethanol composite locking material. In an experimental environment that simulates rainfall (simulated by dripping water through a rubber‐tipped burette), when the ambient humidity reaches a specific threshold, water infiltrates the interface between the seed and the actuator groove, which dissolves the PVP, resulting in a loss of mechanical bonding between the two. Under the effect of magnetic force, the seeds begin to slide slowly along the groove. When the lock material is completely dissolved, the two sides of the magnetic poles are instantly closed, and the seeds are ejected at high speed under the concentrated effect of the magnetic field. Humidity triggering takes longer compared to the temperature response (85s for the temperature response and 135s for the water response), mainly due to differences in the diffusion kinetics of liquid penetration versus material dissolution. However, both were able to trigger magnetic energy release via environmental signals, which validates the actuator's ability to achieve multimodal environmental triggering.

### 2.2 Rheological Properties of Magnetic Inks and Structural Optimization

**Figure 2:**
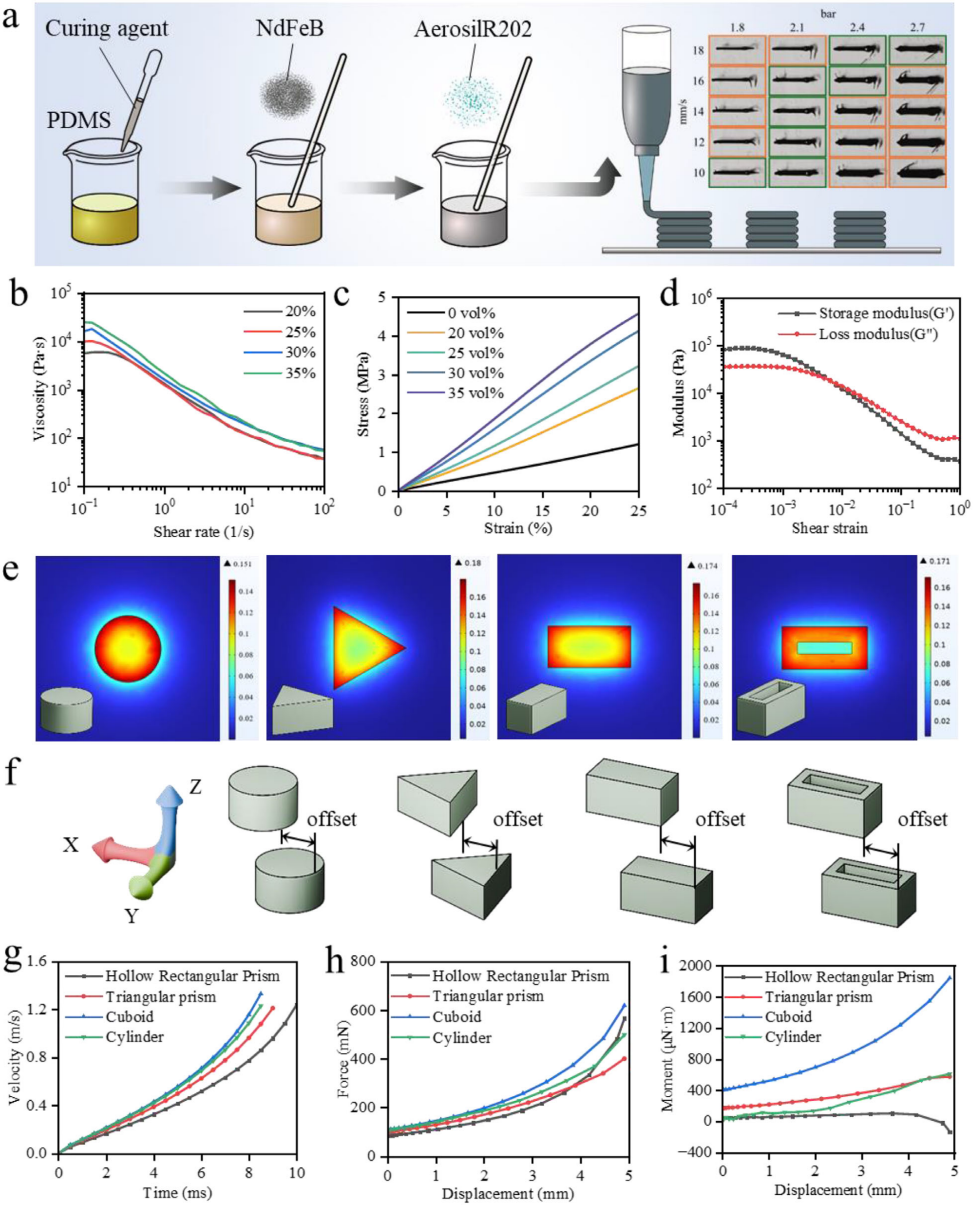
Rheological properties of magnetic inks and structural optimization of ESUs. a) Schematic diagram of PDMS‐based magnetic ink formulation and DIW printing process. b) Effect of NdFeB particle volume fraction (20–35 vol%) on the shear thinning behavior of the ink (shear rate: 0.1–100 s^−1^). c) Stress–strain curves of inks with different NdFeB volume fraction inks. Stress–strain curves of inks with Young's modulus increasing linearly with particle content. d) Viscoelastic properties of 30 vol% NdFeB inks: solid‐liquid transition between G′ and G″ at critical shear strain (0.006). e) Simulation of flux density distribution of the four types of storage units, namely cylindrical, cuboid, triangular prism, and hollow rectangular prism. f) Schematic representation of the offsets defined when the ESUs are attracted to each other. g‐i) Dynamic response of the ESUs at a lateral offset of 3 mm.

Rheological properties of magnetic inks and structural optimization is the key foundation for bionic actuators to achieve high‐precision manufacturing and efficient stored magnetic energy. **Figure** **2**a shows the formulation and printing process of magnetic ink. First, the PDMS matrix was prepared by adding curing agent at a mass ratio of 10:1 and mixing well. Next, NdFeB particles were added to the mixed PDMS and mixed thoroughly with a pharmaceutical spoon, and then fumed silica was added to adjust the rheological properties. As shown in Figure S2 (Supporting Information), a slurry ratio of 0.105:1 for the mass ratio of fumed silica to the PDMS kit was selected through the rheological property test. Subsequently, the formulated magnetic ink was transferred to the print syringe for DIW. The DIW printing process is shown in Movie S1 (Supporting Information). A 0.41 mm extrusion head was used for printing, and during the printing process, the diameter of the lines increased as the extrusion pressure increased or the printing speed decreased. Experiments have shown that insufficient extrusion pressure can cause minor accumulation of solid particles within the nozzle, leading to fluctuations in nozzle pressure and consequently, uneven extruded lines. At this point, by adjusting the print speed can not improve the uneven lines of the phenomenon; and when the extrusion pressure is too large, the required print speed is too high, excessive increase in extrusion speed will result in the line in the bends and bends in the poorly formed. After line analysis, the green box labeled parameters can obtain better printing results. Further, this study adjusted different line spacing to investigate its effect on the printing effect (Figure S3, Supporting Information), and finally produced samples with fewer defects (Figure S4, Supporting Information). Limited by material properties, structural complexity, and time pressure, this study used an empirically based method to determine the printing parameters, but still guaranteed uniform stability and structural quality of the printed line. In the future, we will optimise the parameters computationally to improve the printing efficiency and accuracy consistency.

In this study, the correlation between the shear‐thinning behavior of the ink and its printability was revealed by systematically investigating the effect of the volume fraction of NdFeB particles (20–35%) on the ink properties. Figure 2b demonstrates the rheological properties of printing inks with NdFeB particle volume fraction. All inks exhibit significant shear‐thinning behavior, which is attributed to the 3D network structure built by the silicon hydroxyl groups on the surface of SiO_2_ through hydrogen bonding: the hydrogen bonding network is disrupted by high shear stresses, which leads to a sudden drop in the viscosity of the inks. For example, when the shear rate is increased from 0.1 to 100 s^−1^, the viscosity of 35% by volume ink decreases by three orders of magnitude from 24772 to 54.6 Pa·s (Figure 2b), which ensures the stable extrusion of complex structures in the DIW process (Figure 2a). Meanwhile, the hydrogen bonding network can be rebuilt quickly after extrusion, enabling the extruded lines to resist gravity and maintain morphological stability. It is noteworthy that although the viscosity of the high volume fraction (35%) ink was higher than that of the low volume fraction (20%) ink at low shear rates (24772 and 5612 Pa·s, respectively), the difference in viscosity was drastically reduced at high shear rates (100 s^−1^) (54.6 and 36.6 Pa·s, respectively). These results highlight the critical balance between pressure and speed to ensure printing precision.

Further, the mechanical property test results further revealed the modulation law of the particle volume fraction on the material stiffness (Figure 2c). It was found that the introduction of NdFeB particles significantly suppressed the hyperelasticity characteristics of the PDMS matrix: when the volume fraction of NdFeB particles was increased from 0% to 35%, the composites exhibited an approximately linear mechanical response in the 0–10% strain interval. The Young's modulus, calculated by direct measurement, linearly increased from 4.9 to 17.1 MPa with the increase of the particle volume fraction; for scenarios involving large strains, the Ogden model will be introduced in subsequent studies to improve the accuracy of the mechanical analysis. Meanwhile, the radius of curvature at fracture increases dramatically with the increase of volume fraction. When the volume fraction reaches 35%, the radius of curvature at fracture has reached ≈1.5 mm, which is already unfavorable for the structural design of the stored magnetic energy actuator (Figure S5, Supporting Information). So in this study, we chose the ink with 30% volume fraction of NdFeB particles for the fabrication of the stored magnetic energy actuator. The viscoelastic analysis (Figure 2d) further verifies the dynamic properties of the ink with 30% volume fraction of NdFeB particles. When the shear strain is 0.006, the energy storage modulus (G′) intersects with the loss modulus (G″), which indicates that the ink exhibits a solid‐state behavior (G′ > G″) at low strains to ensure the integrity of the printed structure. Whereas, at high strains (> 0.006) it exhibit liquid behavior (G″ > G″), which facilitates extrusion and interlayer bonding.

The geometrical design of the energy storage unit (ESU) is the core of realizing energy‐efficient magnetic coupling and stable dynamic response. The magnetic field distribution characteristics and dynamics performance differences of four heterogeneous structures, namely, cylinder, cuboid, triangular prism and hollow rectangular prism, are systematically investigated through finite element simulation and parametric dynamics simulation (Figure 2e–h). The simulation of magnetic flux density distribution shows (Figure 2e) that the sharp geometrical features significantly enhance the magnetic field concentration effect. The magnetic flux densities at the right angles/prisms of cuboid and triangular prisms reach 0.174 T and 0.18 T, respectively, which are enhanced by 14.6%‐18.5% compared with that of cylinders (0.151 T). This phenomenon stems from the focusing effect of the sharp edges on the magnetic inductance, which allows the magnetic field energy to be applied to the target area more efficiently. It is worth noting that the hollow rectangular prism, due to its internal hole structure, allows the magnetic lines of inductance to penetrate in both directions (external boundaries and internal hole walls) to form a uniformly distributed magnetic field (maximum flux density of 0.175 T), combining high field strength with multi‐directional drive potential.

In practice, due to the influence of structural or positional fixation, it is difficult for two ESUs to achieve perfectly aligned attraction or repulsion motion, and there is bound to be lateral offset. Therefore, its interaction performance under various relative offset states is what determines the actual performance of the ESUs. Here, we evaluate the performance of different ESUs under actual offset conditions. As shown in Figure 2f, the lower ESU is fixed in the experiment, so that the upper ESU generates a relative offset along the defined X‐direction (the Y‐direction is kept perfectly aligned, and the Z‐direction spacing is fixed at 5 mm). By constraining the X‐ and Y‐direction degrees of freedom of the upper kinematic ESU, its performance under different X‐direction offsets and the same Z‐direction spacing was characterized (Figure 2g–i; Figures S6–S9, Supporting Information). Further, the mutually attractive kinetic behavior of the upper unit was parametrically characterized over a range of X‐direction offsets (offset = 0–3 mm). The results show that the cuboid maintains the highest magnetic attraction force (619 mN) and velocity stability at 3 mm offset, and its maximum torque reaches 1.84 mN·m, which is 162% higher than that of the triangular prism (<0.7 mN·m). The high torque originates from the strong magnetic field gradient in the right‐angle region of the cuboid, which can generate a self‐aligning moment during deflection, effectively correcting positional deviation and reducing energy loss. This dynamic self‐correction mechanism makes it significantly superior in directional high‐speed drive scenarios, especially for tasks requiring high motion accuracy.

In contrast, the hollow rectangular prism ESU shows excellent adaptability in multi‐degree‐of‐freedom motions due to its internal hole structure that creates a more uniform magnetic field distribution (Figure 2e). Although its magnetic attraction force (566 mN) and instantaneous velocity are slightly lower than that of the cuboid, it shows more robustness in complex paths (e.g., non‐planar trajectories or multi‐obstacle environments). After analysing the magnetic field distribution and dynamics, the cuboid and hollow rectangular prism are selected as the optimal ESUs, each with its own advantages. The cuboid is suitable for directional high‐speed driving scenarios, and its sharp edges' magnetic field concentration effect and high torque characteristics ensure efficient energy transfer and precise control. The hollow rectangular prism, with its uniform magnetic field distribution and multi‐directional driving potential, is more adaptable to complex environments and meets the multi‐degree‐of‐freedom requirements of adaptive systems. This synergistic design strategy not only solves the stability problem of the ESU under offset conditions, but also provides flexibility for the actuator to be used in a variety of tasks. It should be noted that the current study has improved the offset tolerance by optimising the ESU geometry. In the future, we will further explore the combination of the segmented magnetisation technique with multi‐material DIW printing to achieve the design of magnetic domain gradients inside ESUs to improve the alignment accuracy and expand their functional applications.

### 2.3 Magnetic Energy‐Mediated Seed Ejection Mechanisms

**Figure 3:**
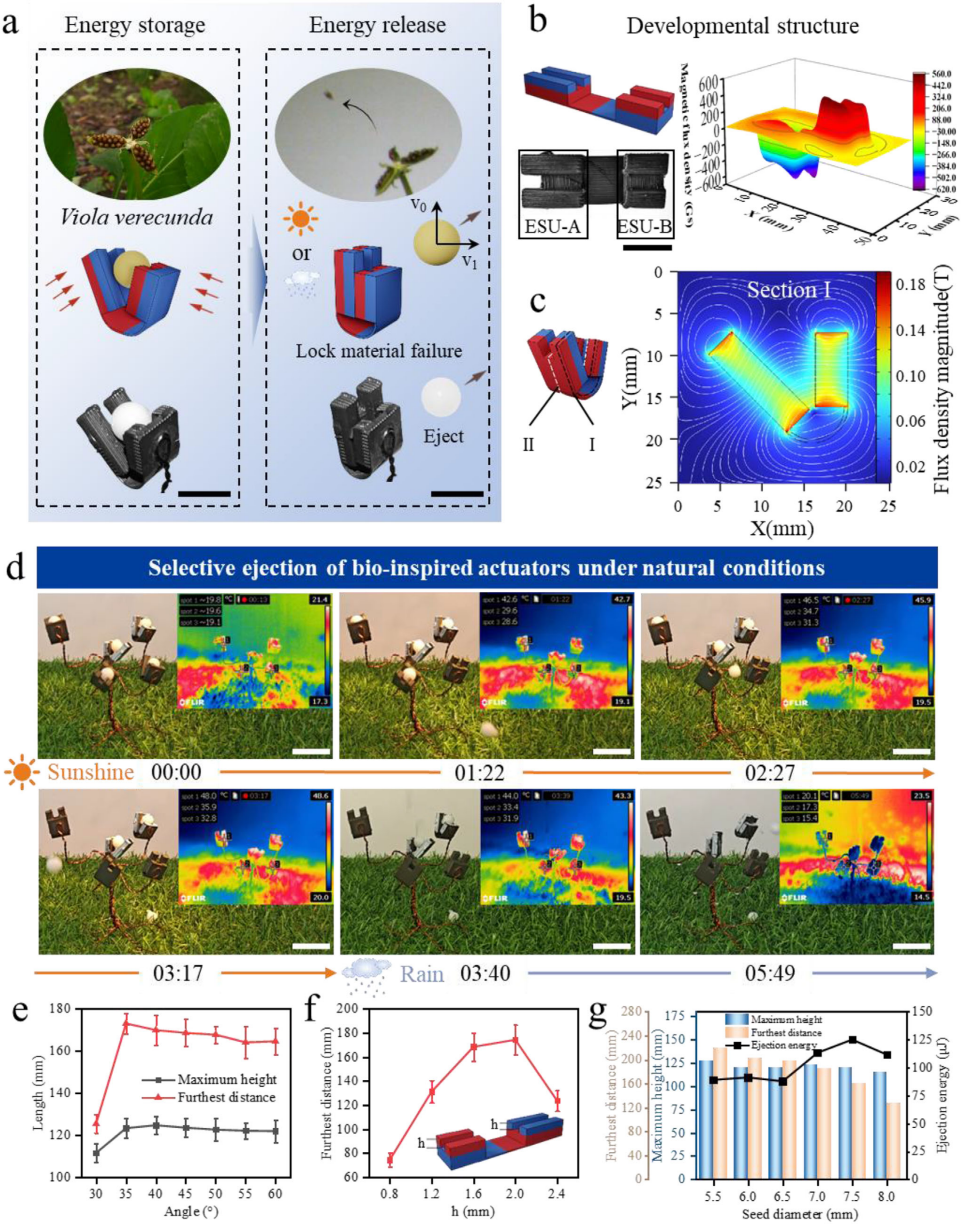
Storage magnetic energy ejection actuators mimicking *Viola verecunda* seed pods: structural design, environmental response and performance optimization. a) Schematic of the asymmetric stored magnetic energy actuator structure. Scale bar: 10 mm). b) Actuator unfolded state and magnetic field distribution (blue: N‐pole, red: S‐pole). Scale bar: 10 mm. c) Cross‐section magnetic flux density simulation (peak 0.205 T). d) Selective ejection triggered by temperature (yellow light heating, 85 s response) and humidity (simulated rainfall, 145 s response). Scale bar: 25 mm. e) The effect of clamping angle (30–60°) on the distance and height of seed ejection. f) The modulation of the ejection distance by the height above the groove *h* (0.8–2.4 mm). g) Ejection performance of different seed diameters (5.5–8.0 mm).

Inspired by the efficient ejection seeding mechanism of *Viola verecunda* seed pods, a stored magnetic energy ejection actuator based on magnetic energy attraction was designed in this study. The ejection actuator consists of a plurality of cuboid‐like ESUs spliced together. These units are arranged on both sides of the groove and are connected to form an integrated structure by transverse connecting pieces at the bottom. The seeds borrowed a natural responsive material, which was fixed in the center plane of the grooves between the ESU‐A and ESU‐B of the actuator (**Figure** **3**a). When the external conditions change, the responsive material gradually fails. After the failure of the response material, the seed and the actuator slide relative to each other, and the system is destabilized when it reaches a specific node, thus realizing transient ejection, and the workflow integrates the failure of the material and the destabilization of the system to complete the rapid release of energy.

The unfolded structure of the ejection actuator (Figure 3b; Figure S11, Supporting Information) has two key features: first, the lengths of the ESUs on both sides are asymmetric, where ESU‐A is significantly longer than ESU‐B. This asymmetric design enhances the magnetic force between the units on one hand, and on the other hand, the longer ESU‐A is able to provide horizontal thrust to the seed, which results in a higher horizontal initial velocity. This initial velocity, when synthesized with the motion velocity in the other direction, not only changes the motion trajectory of the seed, but also significantly enhances its synthesized velocity. Therefore, the asymmetric geometry of the structure is designed to achieve an oblique throwing trajectory through velocity vector synthesis, an optimisation based on functional requirements rather than the basis magnetic coupling model. Second, the middle of the actuator is equipped with a transverse groove. Due to the uneven distribution of the magnetic force of the ESU, when there is no groove, the seeds are subjected to uneven force, deviating from the intended ejection route and prolonging the closing time and wasting magnetic energy. The groove acts as a guide to significantly improve the ejection stability and actuator performance repeatability. Epimagnetic analysis shows that the maximum flux density of the actuator can reach – 620 Gs, the flux density on both sides of the groove is higher than that at the groove, and the position of the magnetic field reversal is consistent with the bending position of the two‐unit joint, which is in line with the design expectation. The flux density in the middle area of the extra square structure on one side is nearly zero, indicating that although the actuator structures are connected, the connection does not have a significant effect on the flux density.

In view of the large difference in the magnetic flux density distribution between different sections due to the heteromorphic design of the actuator, the magnetic field distribution of the actuator under the attitude of mutual attraction between the two stored magnetic energy units during operation is simulated using simulation software. Taking sectionI as an example (Figure 3c), the higher flux density is concentrated in the corners, the plane is relatively small, and the overall maximum flux density reaches 0.205 T. In this attitude, the high density of magnetic inductance below the two storage units indicates that the attraction from below is the main driving force for the start of the motion, and the flux density at section II is weaker than that at section I (Figure S12, Supporting Information).

The ejection process of the actuator is mechanically analyzed, and Figure S13 (Supporting Information) represents the whole ejection process. The whole system is divided into three steps. The first is the steady state of the system, as shown in Figure S13a (Supporting Information), when the seed is stationary and the forces are balanced:

(1) $$F_{combine}=0$$

As the responsive material fails, the seed begins to slowly slide inside, as shown in Figure S13b (Supporting Information). According to the interaction force of the magnetic dipole:

(2) $$F_{m}=\frac{3\mu_{0}}{4\pi r^{4}}\left[\left(\vec{m_{1}}\cdot \vec{m_{2}}\right)-3\left(\vec{m_{1}}\cdot \hat{r}\right)\left(\vec{m_{2}}\cdot \hat{r}\right)\right]$$

where*F_m_* represents the interaction force between two magnetic dipoles; ***r*** represents the mutual distance, $\vec{m_{1}}$, $\vec{m_{2}}$ denote the two magnetic dipole vectors; $\hat{r}$ represents the unit vector from one magnetic dipole to the other.

As the seed slides relative to the actuator, the change in distance results in a change in magnetic suction, at which point the seed force is based on Newton's second law:

(3) $$F_{combine}=ma$$

The combined forces of thrust, gravity and friction on the seed give the seed a slow acceleration. This acceleration drives the seed to start moving progressively faster, as in the state represented in Figure S13c (Supporting Information), and this eventually gives the seed a vertical upward velocity*v*_0_. When the seed slides to a specific node, the drastic change of the system structure triggers the instability of the overall structure, as shown in Figure S13d (Supporting Information). The magnetic energy E_m_ originally stored inside is rapidly converted into the kinetic energy of the seed, and according to the law of energy conservation, part of the magnetic energy is converted into the kinetic energy of the seed, while the other part is lost under the collision of the units on both sides, in accordance with the kinetic energy theorem:

(4) $$E_{m}=\frac{1}{2}mv_{1}^{2}+E_{loss}$$

And the final velocity of the seed*v* is the combined velocity of *v*_0_ and *v*_1_ in the direction of tilt upward. The final stage of the seed is the flight stage, when the seed is only acted by gravity and smaller air friction, which makes the seed finally show an oblique upward throwing motion externally, completing the whole ejection process.

The selective response of the actuators to natural conditions was experimentally verified (Figure 3d; Movie S2, Supporting Information). An actuator cluster consisting of five actuators was constructed with one seed immobilized in each actuator. Three of them were immobilized by phase change wax with a melting point of 37 °C, which was triggered by the thermal response, and were arranged according to the distance from the heat source for studying the relationship between the heating efficiency and the ejection time. The other two were immobilized by PVP ethanol solution, which was triggered by the water response. Here, the yellow light simulated the sunlight, and the seed closest to the sunlight (≈42 °C) was ejected at 82 s; the second seed was ejected at 147 s; and the last heat‐responsive seed was ejected at 197 s when the water‐responsive actuator was still stable. The light was withdrawn and the simulated rainfall started at 220 s, and the water‐responsive actuator ejected the seed at 349 s, which was the end of the ejection process. The experiment confirms that: the specific response actuator is not interfered by other environmental changes, which ensures the stability of the trigger; different thermal efficiencies have a significant effect on the ejection trigger, but all of them are able to complete the ejection process in the end.

The condition and structure of the actuator itself had a significant effect on the ejection performance. The performance characterization of the actuator clamping angle (Figure 3e) showed that, when the actuator was fixed at 90 mm above the ground and the 7 mm diameter seeds were ejected, the ejection was not effective at an opening angle of 30°; the difference in the maximum ejection distance and the maximum height was not significant between 35° and 60°. Changing the actuator size (Figure 3f) increases the height above the groove, ***h***, from 0.8 to 2.4 mm. The ejection distance is only 74.5 mm at 0.8 mm, and increases rapidly but slowly with increasing height ***h***. the maximum value of 174.5 mm is reached at 2 mm, and then the distance decreases dramatically at 2.4 mm, which is mainly due to the fact that the connecting plate is restrained by the units on both sides, and the connecting plate dissipates a large amount of magnetic energy when the height ***h*** exceeds the limit. Considering that the effect of increasing the height on the performance is slowing down, a print height of 2 mm is appropriate.

The actuator generalization study (Figure 3g) used a 2 mm height ***h*** actuator to eject seeds of different diameters. The results showed that the best overall performance was achieved when the seed diameter was 5.5 mm. With the increase of seed diameter, the maximum height decreased from 127 to ≈115 mm, with small changes; the farthest distance decreased from 220 to 128 mm, indicating that the force on the seeds was tilted upward, the vertical velocity changed little, and the horizontal velocity was slowed down, which might be related to the different forces on the seeds of different diameters. The maximum height and the farthest distance were the lowest when the particle size was 8 mm, but due to the difference in the quality of seeds with different particle sizes, according to the kinetic energy calculation, the 7.5 mm diameter seeds obtained the maximum kinetic energy, which corresponded to the optimal energy transfer efficiency of the actuator.

In summary, the actuator in this study is capable of triggering high‐speed ejection under specific environmental thresholds (e.g., specific temperature and humidity conditions) by means of the instantaneous release of attractive or repulsive forces between magnetic poles. This strategy has important potential applications in damaged ecological areas such as fires, desertification or mining wastelands: the ejection actuator can be pre‐buried in the soil, and when the environmental conditions reach a predefined threshold, the actuator automatically ejects a capsule carrying plant seeds or microbial spores, thus allowing for precise dispersal.

### 2.4 Bionic Gripping Actuator

**Figure 4:**
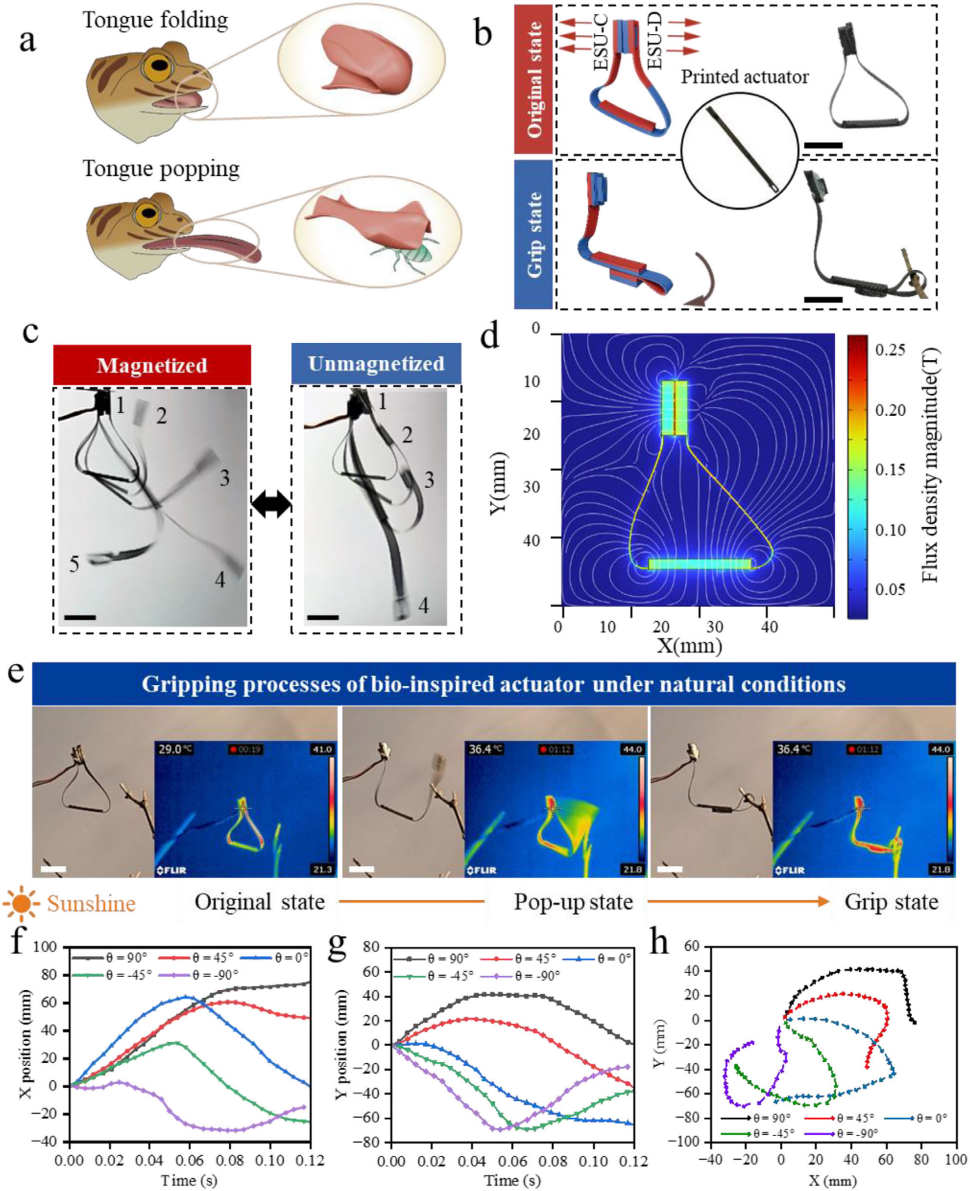
Gripping actuator imitating frog tongues: multimodal actuation and dynamic response mechanism. a) Schematic diagram of the bionic principle of frog feeding on insects. b) Structural design and workflow of the stored magnetic energy gripping actuator (blue: N‐pole, red: S‐pole). c) Comparison of the trajectories of magnetized and unmagnetized actuators. d) Simulation of the flux density distribution in the stored state. e) The whole process of gripping a branch of a tree by the actuator under the high‐temperature triggering (simulation of yellow light). f‐h) Effects of different placement angles (θ = −90° to 90°) on the X/Y displacement, velocity vector and motion trajectory of the gripping endpoint. Scale bar: 10 mm.

In nature, the energy burst system is not unique to plants, and many animals have evolved similar mechanisms, the frog tongues being a classic example. As shown in **Figure** **4**a, frogs are able to capture insects within 0.07 s by means of a folded tongue hidden in their mouths, which can be rapidly ejected and retracted by a special muscle design. When hunting, the frog tongues also folds longitudinally to hold the prey, which increases the adhesion area with the insect, and also has the function of gripping.^[^ ^45^ ^]^ However, the current frog tongue bio‐inspired soft actuator only allows for simple bending and cannot provide a firm grip on the target.^[^ ^46^ ^]^ Here, the frog tongues is used as a bionic prototype, and combined with the previous kinetic analysis of the repulsive behavior of the ESU, a stored magnetic energy gripping actuator that imitates the frog tongues to catch prey is developed, and the specific structure is shown in Figure S14 (Supporting Information). The actuator can not only be quickly ejected using magnetic energy, but can also be converted into an attraction behavior by its own motion in order to grab objects.

The magnetic distribution and operating state of the whole gripping actuator is shown in Figure 4b. As can be seen from the color schematic of the magnetic poles, the actuator adopts a complex folding magnetization method. In actual operation, the ESU‐C and ESU‐D are initially mutually exclusive and fixed by the responsive material. In case of external environmental stimuli, the connection between the two ESUs fails rapidly, and the hollow rectangular prism unit drives the connecting piece to fly out. When moving in the air, if intercepted by an object to be grasped, the ESU will move around the object in a curved motion, and finally realize the attraction between the units.

Unlike the seed ejection actuator, which exhibit only passive mechanical relaxation in the absence of a magnetic field. The gripping actuator has a tendency to move forward and downward in its overall structure due to the influence of gravity, so it is necessary to differentiate whether its gripping behavior is driven by magnetic energy or gravity. Figure 4c shows the different motion states of the unmagnetized and magnetized actuators, where the left side of Figure 4c shows the complete motion of the gripping actuator after it is ejected after magnetization, and the right side of Figure 4c shows the motion of the un‐magnetized actuator after the connection is dislodged.

As can be seen on the left side of Figure 4c, the trajectory of the hollow rectangular prism ESU is nearly horizontal after it is ejected. As the motion proceeds, it moves diagonally downward due to its own weight and the pull of the ESU below. This indicates that the ESU with a certain initial kinetic energy enables the actuator to move to the right side, expanding the gripping space. Figure 4c Position 4 on the left shows the furthest position that the gripping actuator can reach. At this point, the ESU consumes some of its kinetic energy to keep the connecting unit stable, but it still has a high speed and moves rapidly to the left, forming a reverse C‐shape. During the rapid movement interval of the ESU, it has a certain gripping ability when encountering obstacles. The right side of Figure 4c shows that the unmagnetized ESU falls rapidly to the lowest end, and its motion is basically converted by gravitational potential energy, with a small horizontal range of motion and no horizontal long‐distance gripping capability.

After determining the structure, the magnetic field distribution of the overall structure under the working condition was analyzed by epimagnetic analysis (Figure S15, Supporting Information) and simulation. As shown in Figure 4d, the two ESUs repel each other when the actuator is in operation. From the distribution of magnetic lines of inductance and the flux density cloud diagram, it can be seen that the magnetic lines of inductance converge at the repulsion place of the two ESUs, and the cloud diagram shows that the flux density here is larger, indicating that the repulsion of the two sides of the storage units is effective. The magnetic inductance of the lowermost ESU mostly converges at the connecting units on both sides, indicating that in practice, the magnetism of the lowermost ESU has less influence on the repulsive behavior of the units above it.

The gripping behavior of the gripping actuator in hot weather was investigated (Figure 4e; Movie S3, Supporting Information). A yellow light was used to simulate sunlight, and phase change wax with a melting point of 37 °C was used as the thermo‐responsive material between the two ESUs, which was fixed to the connecting piece of the other side of the storage unit through the gap in the middle of the hollow rectangular prism ESU. After exposure to light, the phase change wax fails and the hollow rectangular prism unit quickly pops out, and the connecting piece encounters an obstacle and moves around the branch in a curved motion, and finally moves to the bottom of the elongated rectangular unit, where the attraction force comes into play and closes the two units, completing the gripping. The whole process took ≈72 s. From the viewpoint of the movement process, first, thanks to the design of the two vertical connecting pieces on the ESU, after the failure of the phase change wax, the majority of the wax was fixed on the other ESU, which did not affect the movement of the ejected unit. Second, the maximum ambient temperature was only 44 °C, which is a common level of high temperature in nature. The surface temperature of the ejected part was 36.4 °C before ejection, and the internal temperature changed to 36.3 °C after ejection, indicating that the hollow rectangular prism design effectively mitigated the problem of uneven heating between the inside and outside of the actuator and ensured a quick and responsive gripping of the actuator.

Both the structural dimensions and the placement angle of the gripping actuator have a significant effect on its kinematic behavior. Here, by changing the height ***h*** of the ESU (Figure S15, Supporting Information), it was found that the gripping actuator with ***h*** of 3.25 mm had the largest gripping range (Figure S16, Supporting Information). Meanwhile, the horizontal ejection direction of the ejected ESU was assumed to be θ = 0°, with counterclockwise rotation being positive and clockwise rotation being negative. The ejection angles of the actuators 90° to – 90° are characterized and the results are shown in Figure 4f,g. Figure 4f shows the X‐coordinate of the endpoint of the ESU over time. The actuator endpoint with an ejection tilt angle of 0° has the fastest initial velocity in the X‐direction, but falls back quickly at 0.06 s. The actuator with a tilt angle of 90° ends up with the largest range of movement in the X‐direction. Figure 4g shows the Y‐coordinate of the endpoint of the ESU over time. As the angle of θ changes from negative to positive, the time for the endpoint of the gripping actuator to reach the lowest point of the Y‐position is gradually shifted back. Figure 4h presents the motion path, velocity and direction of the endpoint of the gripping actuator at different angles. It can be seen that the gripping actuator with a placement angle of 90° has the largest motion range, which indicates that the gripping area of the actuator is the largest when the ESU is ejected upward. As the angle changes to a negative value, the integrated motion speed of the actuator increases, and when the angle of is −90°, the gripping actuator can even realize self‐gripping motion without gripping the object.

The device is designed with a folded magnetised structure, which can be quickly unfolded and ejected after environmental triggers, and use the magnetic attraction to complete the target capture, which has important application potential in field monitoring, disaster rescue and debris detection. For example, in the field of biodiversity survey, the gripper actuator can be deployed in forest or wetland environments to assist researchers in collecting samples by triggering the grapple action through temperature or humidity changes. Or in disaster relief scenarios (e.g., earthquake or collapse sites), the gripper actuator can be integrated into small exploration robots, triggered by environmental variables, to locate and grasp key items under obstacles.

### 2.5 Logic Gated Jump Actuator

**Figure 5:**
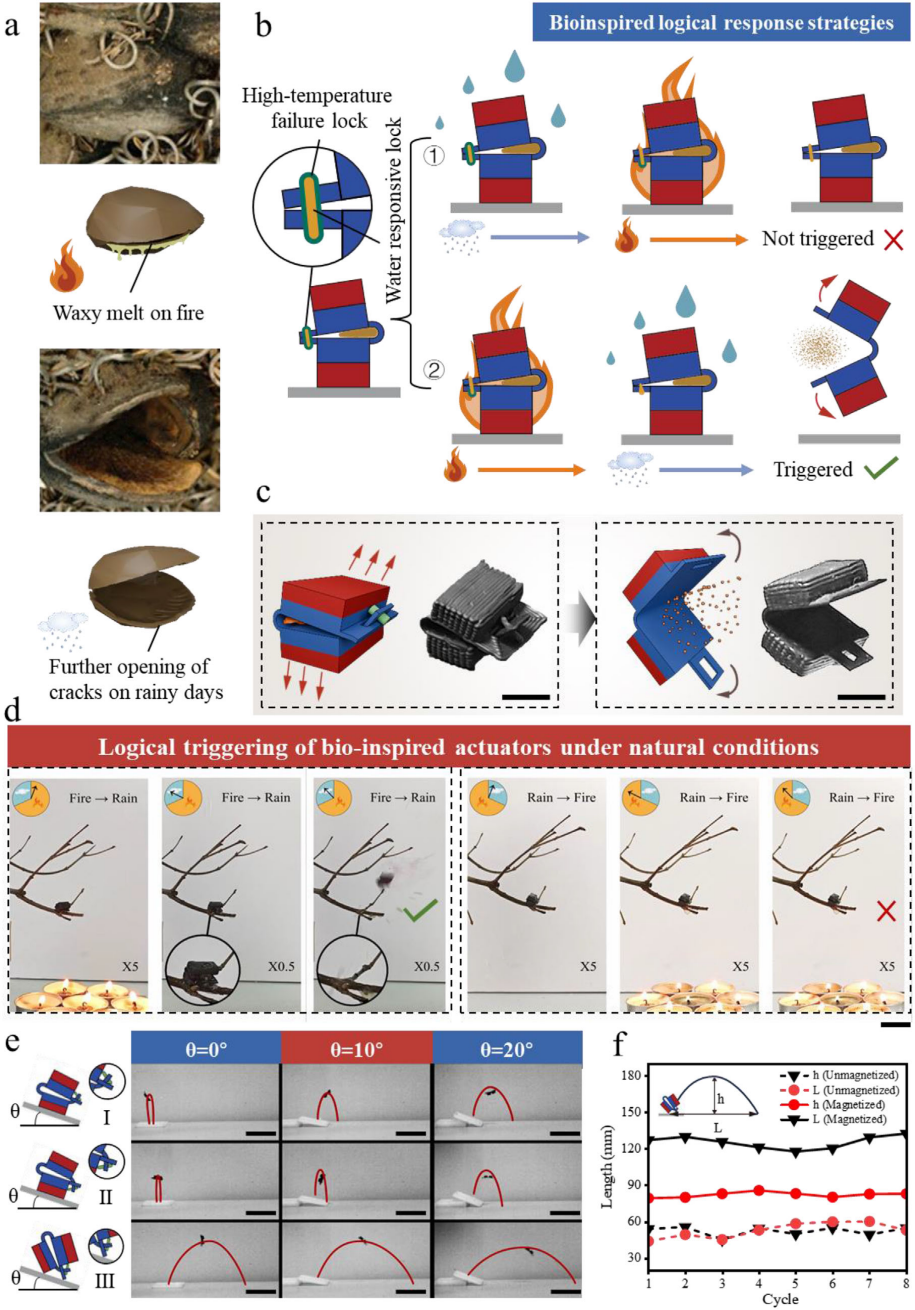
Logic‐responsive actuators modeled after *Banksia ericifolia*: structural design, sequential triggering, and dynamic performance optimization. a) Seed release mechanism in the seed pods of *Banksia ericifolia*.^[^ ^48^ ^]^ b) Actuator “locks ” structural design and logic gating principle. c) Folding state and bouncing workflow of the magnetic energy storage jump actuator. Scale bar: 5 mm. d) Influence of the sequence of environmental conditions on the triggering behavior (left: fire→rainfall successful triggering; right: rainfall→fire no response). Scale bar: 20 mm. e) Effects of different placement angles (forward, inverted, and inclined) on the jumping trajectory. Scale bar: 50 mm. f) Comparison of jumping performance between magnetized and unmagnetized actuators.

The endemic Australian *Banksia ericifolia* has a unique seed pod configuration. The seeds are not self‐seeded, but are triggered by a series of external changes. As shown in **Figure** **5**a, the outside of the seed pod opening is covered with a self‐produced wax that is unaffected by regular outside temperatures. During a forest fire, the wax on the outside of the seed pods melts, the plant fibers in the openings deform slightly due to drying, and the seed pods open up. Subsequently, when the moisture content in the air increases and the plant senses a humid environment suitable for seed germination, the moisture induces a deformation of the double‐layered fiber structure inside the seed pod, which gradually opens up and eventually releases the seeds.^[^ ^47^, ^48^ ^]^

Drawing on the principle of multifactorial judgment response of *Banksia ericifolia* seed pods, we designed a jump actuator with a natural‐conditional logical response. The natural condition logical response is defined as the ability to complete the scheduled triggering after the appearance of the set natural conditions in sequence, no movement or response before the appearance of all the conditions, and can only be triggered strictly in the order of the conditions. As shown in Figure 5b, the jump actuator is held in place by a “locks”, which is made of a combination of high temperature phase change wax and PVP ethanol solution, and is manufactured as shown in Figure S17 (Supporting Information). This jump actuator is not responsive when first experiencing rainfall, and then experiencing a forest fire; Instead, it can be triggered to respond logically by experiencing a forest fire followed by rainfall to jump and spread spores.

The structure of the stored magnetic energy jump actuator with logic response is shown in Figure S17 (Supporting Information). The structure contains two rectangular ESUs and a connecting piece in the middle, which is used to connect and position the two units when folded in reverse. The two ends of the actuator are slender slots and a long strip with a hole in the middle of the strip. In practice (Figure 5c), the structure is first folded in the reverse direction, and the long strip is inserted into the slot and secured with a “locks”. By virtue of the special structural design, the repulsive force of the two ESUs is transformed into the shear force of the structure on the “locks”. When the external environment stimulates the “locks” and causes it to fail quickly, the whole structure quickly pops open under the repulsive effect of the ESUs due to the great magnetic flux density at the two storage units and their mutual repulsion (Figure S19, Supporting Information). This movement causes the whole structure to deform dramatically for a short period of time, and the lower part of the structure in contact with the ground pushes the whole structure to bounce upwards while spreading the internal spores. Figure 5d and Movie S4 (Supporting Information) demonstrate the logical response of the jump actuator in natural environments. The figure is divided into left and right parts, with the left side showing the natural environment of a fire followed by rain, and the right side showing the environment of rain followed by a large fire, and the duration of each condition is strictly the same on both sides. It can be clearly seen from the figure that the whole actuator completes the triggering process in ≈75 s after the natural environment change of fire followed by rainfall on the left side, while on the right side, even though the same condition occurs, it is not in the same order and the actuator cannot be triggered. The “spores” used here are replaced by purple potato pigment powder, which is used as a tracer to simulate the release of spores. A visual demonstration of the successful release of the pigment powder under magnetically triggered conditions.

Differences in natural environments determine the variety of actuator placement. In this study, we investigated the jump performance of actuators in forward, inverted, and inclined placements, and set up different angles in each placement to investigate the effect of placement in natural environments. The results are shown in Figure 5e and Movie S5 (Supporting Information), and the red line indicates the actuator jumping path. As can be seen from the figures, both forward placement (I) and inverted placement (II) can only realize in situ landing and taking off, and the performance of inverted placement is weaker than that of forward placement, which is due to the asymmetric design of the edge structure of the left and right sides of the actuator. In the initial stage of bouncing, when the actuator opens, its edges will contact with the ground, and the edges with different geometrical configurations have slight differences in the support strength and angle of the actuator, which ultimately leads to different jumping postures and trajectories of the two. With the increase of tilt angle, both of them have a tendency to move forward, but the performance of inverted placement is still not as good as that of forward placement. The performance of inclined placement (III) is much better than that of I and II, not only the jump height is higher, but also the forward jump distance is considerable, and its specific jump posture is shown in Figures S20 and S21 (Supporting Information). With the change of angle, the jump height ofIII decreased slightly, but the jump distance increased rapidly, with the longest distance exceeding 170 mm in the available tests. Compared to current jumping soft robots, the actuators in this study have significant advantages in terms of ease of fabrication and jumping capability (see Figure S22, Supporting Information for details).^[^ ^49^, ^50^ ^]^ Notably, the extended jumping distance of the actuator is not just a kinematic outcome, but also an ecological adaptation strategy. By coupling magnetic energy release with jumping stance, spores are projected beyond competitive microenvironments (e.g., shaded areas under parent plants), significantly increasing the probability of colonization in nutrient‐rich patches.

During actuator bouncing, not only is magnetic energy involved, but the elastic potential energy of the material itself also feeds the motion. Figure 5f shows the results of an eight‐cycle test of the bouncing motion of an unmagnetized and magnetized actuator in an inclined position. It can be seen that the performance of the structurally modified jump actuator is generally stable. When the actuator is not magnetized, the maximum height and the farthest distance fluctuate between 40 and 60 mm; after magnetization, the maximum height of the actuator reaches ≈80 mm, and the farthest distance is more than 130 mm, which not only shows that the magnetization greatly improves the actuator's bouncing ability and highlights the important role of the magnetic energy, but also improves the height of the bouncing by 1.3–2 times, and improves the farthest distance by more than three times. Which indicates that the actuator's bouncing attitude changes with the addition of magnetic energy, and makes it capable of bouncing at a certain total distance. This indicates that the actuator's bouncing attitude is changed after adding magnetic energy, so that it can adjust the bouncing angle under a certain total energy and obtain better bouncing performance.

Through the “logic gate” design, these actuators will only trigger and release the internal load when specific environmental conditions are met in sequence, making them valuable for applications in areas such as smart ecological restoration, environmental monitoring and early warning. Specifically, in forest fire‐prone areas, logic response actuators can be buried in the soil in advance. When a specific sequence of “fire→rainfall” occurs, the actuators will activate the jumping mechanism, releasing fire‐resistant plant seeds or nitrogen‐fixing microbial spores, efficiently accelerating ecological restoration. In flood‐prone riparian zones, if the environmental sequence “persistent high temperature → heavy rainfall” is formed, the actuators will trigger the jumping action, releasing water level sensors or early warning tags to realise real‐time monitoring and early warning of hydrological changes.

## 3 Conclusion

This study proposes a novel bio‐inspired magnetic actuator paradigm that integrates environmental signal‐triggered transient responses, logic gating, and passive energy storage. By combining hard magnetic materials (NdFeB/PDMS) with responsive locking elements (phase change wax, PVP/ethanol), the actuators achieve rapid, autonomous actuation under natural stimuli such as heat and humidity. Utilizing DIW 3D printing and in situ magnetization, the system enables precise control of magnetization and complex geometry fabrication.

The actuators demonstrate excellent performance across multiple functions: seed ejection mimicking Viola verecunda pods, target gripping inspired by frog tongues, and logic‐gated jumping akin to Banksia ericifolia. Finite element simulations confirm that sharp‐edged rectangular units concentrate magnetic flux (0.174 T), while hollow annular prisms enable adaptive motion paths. These innovations offer a foundation for deployable, environmentally responsive soft robotic systems in applications ranging from ecological restoration to autonomous field robotics.

## 4 Experimental Section

### Preparation and Optimization of Magnetic Ink

Dow Corning DC184 two‐component PDMS was selected as the matrix material, and the PDMS prepolymer was mixed with the curing agent in a beaker at a mass ratio of 10:1, with continuous stirring for 5 min until homogeneous. Subsequently, unmagnetized NdFeB particles with a particle size of 5 µm (20–35% by volume) were added and fumed silica (Aerosil 200) was introduced to regulate the rheological properties, and homogeneous dispersion of the particles was achieved by mixing in a ball mill (600 rpm, 10 min). The final ink was transferred to a 10 mL syringe (Nordson EFD HPX 3 5 10CC) for backup.

### DIW 3D Printing and Curing

A homemade DIW 3D printing equipment was used, combined with 0.41 mm (ejection/jumping actuator) and 0.26 mm (gripping actuator) diameter extrusion heads for layer‐by‐layer printing. Printing parameters were optimized as follows: 0.41 mm extrusion pressure of 840 kPa (dispenser output 210 kPa, booster to provide four times the increase in pressure), printing speed of 14 mm s^−1^, layer height of 0.35 mm; 0.26 mm extrusion pressure of 1040 kPa (dispenser output 260 kPa, booster to provide four times the increase in pressure), printing speed of 12 mm s^−1^, layer height of 0.22 mm. After printing, the samples were cured in a vacuum oven at 75 °C for 2 h to ensure the complete formation of the PDMS crosslinking network.

### Preparation and Magnetization of a Stored Magnetic Energy Ejection Actuator—Structure Forming and Magnetization

The cured ejection actuator was folded along the preset connecting piece, wrapped with anti‐sticky paper and placed into a pulsed magnetizer (2.5 T directional magnetic field) for in situ magnetization of the fold.

### Preparation and Magnetization of a Stored Magnetic Energy Ejection Actuator—Temperature‐Responsive Locks Preparation

Simulated seeds were dipped into molten phase change wax (melting point 37 °C), embedded in the actuator grooves and manually secured for 30 s, and thermally‐triggered locks were formed when the phase change wax solidified.

### Preparation and Magnetization of a Stored Magnetic Energy Ejection Actuator—Water‐Responsive Locking Preparation

Mix polyvinylpyrrolidone (PVP K30, Aladdin) with anhydrous ethanol at a mass ratio of 1:1, and place it in a constant temperature oven at 90 °C for 1 h until it was completely dissolved to produce a transparent viscous solution. A small amount of PVP ethanol solution was taken and placed in the air for 1 h, and stirred after part of the ethanol evaporated. Apply the solution on both sides of the ejection actuator and the simulated seed, clamp the seed between the two grooves of the ejection actuator and fix it manually for 1 min. When the ethanol evaporates, the seeds were immobilized, completing the production of a rainfall‐responsive ejection actuator.

### Preparation of Bionic Gripping Actuators

The planar printed structure was folded into an S‐shaped structure, wrapped with anti‐adhesive paper and then magnetized (2.5 T), and the direction of the magnetic poles was based on the design of the alternating repulsion‐attraction distribution. At the end of the magnetization, the side of the ESU that has a protrusion was fixed by gluing it to the copper wire and the other ESU that repels it was manually fixed to it. Add melted phase change wax into the groove of the hollow rectangular prism ESU, hold it manually for 30 s, wait for the wax to solidify, and use the fixation of the protrusion to the groove to make a gripping actuator that responds to hot weather.

### Preparation of Logical “Locks” for Jump Actuators—Calcium Carbide Composite System

Calcium carbide granules (Aladdin) were pre‐crushed by a pulverizer. Mixed with PVP ethanol solution at the ratio of 3:7 by volume and stirred with a medicine spoon for 2 min to form a homogeneous slurry.

### Preparation of Logical “Locks” for Jump Actuators—DIW Printing and Encapsulation

The calcium carbide composite system was molded into 5 mm lines using DIW, which were then dipped into molten phase change paraffin wax (45 °C) for 5 min, removed and cured, and then cut into 3 mm segments to form sequential response logic “locking material”. In addition, it loaded purple potato pigment powder inside the logic response actuator as a tracer to simulate spore release. This design was able to simulate the properties of specific types of spores or particles, thus visualising the release process and dynamic behavior under magnetic triggering conditions.

### Performance Testing and Characterization—Magnetic Flux Density Distribution

The magnetic field strength at the actuator surface was measured using a Epimagnetic distribution measuring instrument (FE‐2100RC). Magnetic field gradient simulation was performed using the steady‐state “mfnc” interface in COMSOL Multiphysics 6.2. The geometric model was a three‐dimensional structure consisting of a pair of N52‐grade neodymium iron boron permanent magnets (where the residual magnetic flux density modulus of different NdFeB particle volume fractions was set as follows: 20% at 0.149 T, 25% at 0.189 T, 30% at 0.212 T, and 35% at 0.238 T), with a spacing of 5 mm between the magnets. surrounded by a cubic air domain with a side length of 20 mm, and gas boundary conditions were applied. The grid employs a mapping partitioning strategy, with a cell size of 0.2 mm set in the edge regions of the permanent magnets. After three iterations of refinement, the grid independence error was less than 1.5%. The relative magnetic permeability of air and permanent magnets were set to 1 and anisotropic 1.05, respectively. The solver was set to steady‐state mode with a relative tolerance of 1 × 10^−3^; the magnetic field gradient ∇B was calculated using an embedded spatial differentiation operator.

### Performance Testing and Characterization—Dynamic response test

A high‐speed camera (MS03130, 790fps) was used to record the ejection process and the trajectory of the grip. An infrared camera (FLIR E40) monitors the temperature triggering process.

### Performance Testing and Characterization—Environmental Adaptability Verification

Sequential triggering logic and passive energy storage stability were tested by yellow light to simulate high temperature and rubber‐tipped dropper (0.1 mL s^−1^) to simulate rainfall.

### Performance Testing and Characterization—Rheological Characterization

A rotational rheometer (Kinexus Pro+) was used to characterize the rheological properties of the PDMS‐based magnetic inks. The formulated magnetic ink was uniformly coated on a 25 mm diameter parallel plate fixture (gap 0.3 mm) and left to stand for 5 min before testing to eliminate the effect of shear history. The shear‐thinning behavior of the inks was analyzed by steady state shear rate scanning (0.1–100 s^−1^, 25 °C), and the apparent viscosity versus shear rate curve was recorded. To assess the viscoelasticity of the inks, dynamic oscillation frequency scans (strain amplitude 0.1%, frequency 0.1–100 rad s^−1^) and strain amplitude scans (0.01–100%, frequency 1 Hz) were performed to determine the intersection of the storage modulus (G′) and loss modulus (G″).

### Performance Testing and Characterization—Mechanical Property Test

The ink was prepared into a standard thickness of 0.6 mm, width of 5 mm, and length of 30 mm long strip sample using DIW printing technology, and uniaxial tensile test was carried out using dynamic thermo‐mechanical analyzer (DMA 303) at a constant rate of 5 mm min^−1^, and the stress‐strain curve was recorded.

## Conflict of Interest

The authors declare no conflict of interest.

## Supporting information



Supporting Information

Supplemental Movie 1

Supplemental Movie 2

Supplemental Movie 3

Supplemental Movie 4

## Data Availability

The data that support the findings of this study are available on request from the corresponding author. The data are not publicly available due to privacy or ethical restrictions.
