# Supplementary material for: Bio‐Inspired Stored Magnetic Energy Actuator with Transient Triggering and Programmable Logic
Source: Adv Sci (Weinh). 2025 Aug 4;12(40):e09683. doi: 10.1002/advs.202509683 (PMC12561206; doi:10.1002/advs.202509683)
Supplement: Supplementary file 1 — Supporting Information [file ADVS-12-e09683-s004.docx]

Supporting Information

**Bio-inspired Stored Magnetic Energy Actuator with Transient Triggering and Programmable Logic**

Chao Xu^1,2,3^, Shuo Jiang^1^, Lu Zhang^4,^*, Xueli Zhou^1,^^[[1]](#footnote-0)^*, Qingping Liu^1^, Luquan Ren^1^

1 Key Laboratory of Bionic Engineering, Jilin University, Changchun 130025, China

2 Liaoning Academy of Materials, Shenyang 110167, China

3 Weihai Institute for Bionics, Jilin University, Weihai, 264207, China

4 College of Construction Engineering, Jilin University, Changchun 130026, China

**S1 Preparation of responsive materials**

**Water-responsive locking preparation:** We selected polyvinylpyrrolidone (PVP) ethanol solution as the water-responsive material through systematic screening. The repeating unit of PVP (Fig. S1a) contains a polar pyrrolidone ring, and its carbonyl (C=O) and amide (N-H) groups are both strong polar groups. Since water and ethanol molecules are also polar, PVP can be stabilized by intermolecular hydrogen bonding with both of them for efficient solubilization. The resulting solution is yellow (Fig. S1b), exhibits excellent fluidity and adhesion, and is stable under environmental factors. We mixed 10 g of PVP K30 powder with 10 ml of anhydrous ethanol and heated the mixture under sealed conditions at 90°C for 1 h. After cooling to room temperature for 1 h, the solution was ready for actuator-seed bonding.

**Screening of temperature-sensitive phase change materials:** In order to realize the precise response to the target temperature and to reduce the interference of environmental factors, phase change wax was selected as the temperature-responsive material in this study. Compared with ordinary paraffin waxes, phase change waxes can achieve the precise control of phase change temperature by adjusting the length of carbon chains or adding eutectic substances. Near the phase change temperature, the phase change wax undergoes a phase change due to heat absorption, and can rapidly soften from a solid to a liquid state, which meets the needs of rapid material failure during the magnetic energy release process. Considering the temperature fluctuation range of practical applications, phase change wax with a melting point of 37℃ was selected as the temperature-sensitive material in this study, and its melting state at the set temperature of 40℃ is shown in Fig. S1(c).


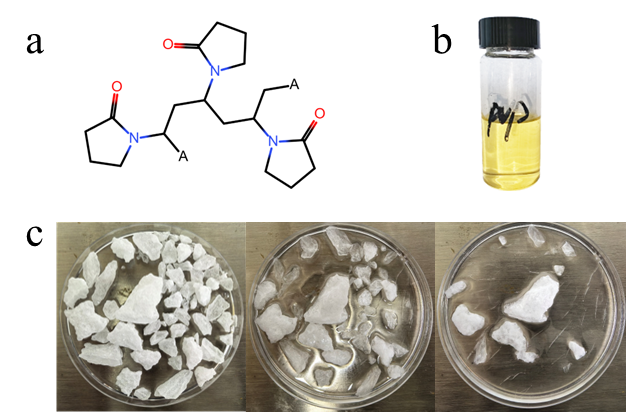


**Fig. S1** Chemical characterization and phase transition behavior of the responsive materials. (a) Chemical structure of polyvinylpyrrolidone (PVP). (b) Appearance and morphology of the ethanol solution of PVP obtained by mixing and dissolving PVP with anhydrous ethanol at a mass ratio of 1:1. (c) Characterization of the heat melting process of phase change wax at 37℃.

**S2 Rheological properties of printed inks**

In this study, the effect of different contents of fumed silica on the ink viscosity was systematically investigated, aiming at screening out the optimal ink ratios suitable for direct writing printing. In the experiments, four kinds of inks with the mass ratios of fumed silica to PDMS kit of 0:1, 0.08:1, 0.11:1, 0.14:1 were prepared, and the rheological performance test results are shown in Fig. S2. The results show that when fumed silica is not added, the effect of NdFeB particles on the rheological properties of the inks is weak. With the introduction of fumed silica, the viscosity of the inks shows a significant increase. When the shear rate is 0.1/s, the viscosity of the slurry with 0.08:1 mass ratio is 940.8 Pa·s, while the viscosity of the slurry with 0.14:1 mass ratio is as high as 66,227.5 Pa·s. With the increase of the shear rate, the viscosity difference of the inks with different fumed silica content is gradually reduced due to the breaking of the hydrogen bonds inside the slurry. When the shear rate reaches 100/s, the viscosities of 0.08:1 and 0.14:1 inks decrease to 43.8 Pa·s and 83.46 Pa·s, respectively, and the actual printing results are summarized in this study, and the final ink ratio of fumed silica to PDMS kit of 0.105:1 is determined, which shows excellent printability and shape retention properties.


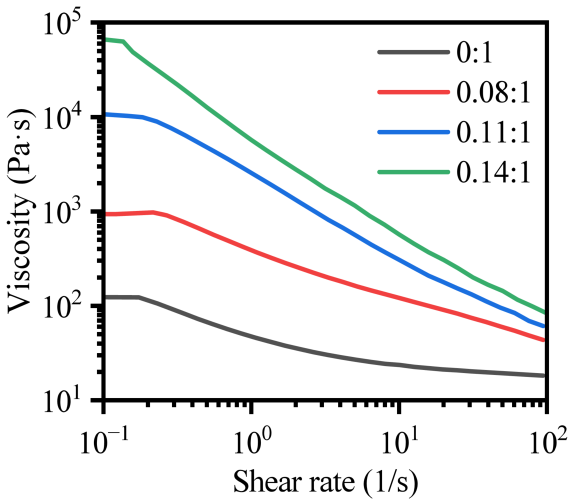


**Fig. S2** Shear rate - viscosity curves of inks with different mass ratios of fumed silica to PDMS two-component materials (0:1, 0.08:1, 0.11:1, 0.14:1).

**S3 Printing parameters for inks**

Print line spacing is a key parameter that influences the printing accuracy and quality of the final sample. This parameter covers two dimensions: planar line spacing and vertical line spacing (i.e., layer height). Under the condition of 0.41 mm print head, the extrusion pressure was set at 2.1 bar, and the print speed was set at 14 mm/s, and the optimization experiments of planar line spacing were carried out (Fig. S3 (a)). Four pitches of 0.3 mm, 0.35 mm, 0.4 mm, and 0.45 mm were selected for testing: when the pitch was 0.3 mm, the neighboring lines were severely extruded and deformed; gaps nearly disappeared, and the excessive stacking significantly affected the height consistency of the samples; and at the pitch of 0.45 mm, the bonding between the lines was loosely connected, which led to a significant decrease in the structural strength. After comprehensive evaluation, 0.35 mm was determined as the optimal planar line spacing.

Layer height (Z-axis stack spacing) plays a decisive role in printability, and improper layer height settings can lead to molding failures due to gravity. In this study, four layer heights of 0.25 mm, 0.3 mm, 0.35 mm, and 0.4 mm were tested at 2.1 bar extrusion pressure and 14 mm/s print speed (Fig. S3 (b)). The experiments show that the 0.25 mm layer height results in excessive compression of the line, causing significant lateral deformation; while the layer height is too large, the bonding force between the layers is insufficient, and with the increase in the number of layers, the vertical structure is gradually deformed, and serious bending occurred by the sixth layer. It is systematically verified that the optimal molding effect can be achieved by setting the layer height to 0.8-0.9 times of the extrusion head diameter, and the layer height of 0.35 mm is finally selected in this study, which effectively guarantees the strength of the interlayer connection and the shape accuracy of the sample. In addition, the SEM images in Fig. S4 visualize the good dispersion of solid particles and the tight connection between the lines, which fully proves that the samples prepared with the optimized printing parameters have a low defect rate, and verifies the accuracy and validity of the parameter selection.


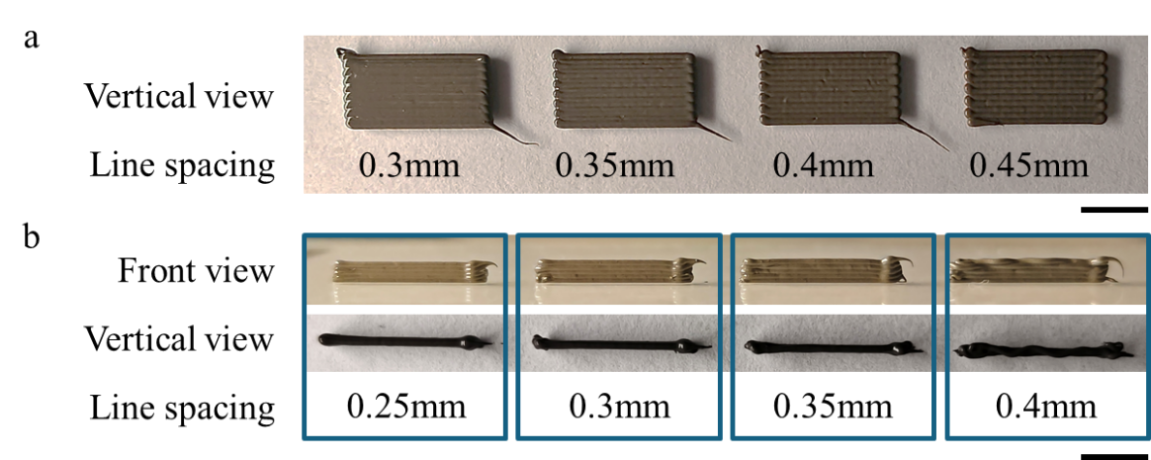


**Fig. S3** Effect of print line spacing on print structure (print parameters: nozzle diameter 0.41 mm, extrusion pressure 210 kPa, print speed 14 mm/s). (a) Influence of planar line spacing on the printed sample. Scale bar: 5 mm. (b) Influence of layer height on the layer bonding performance of the printed sample. Scale bar: 5 mm.


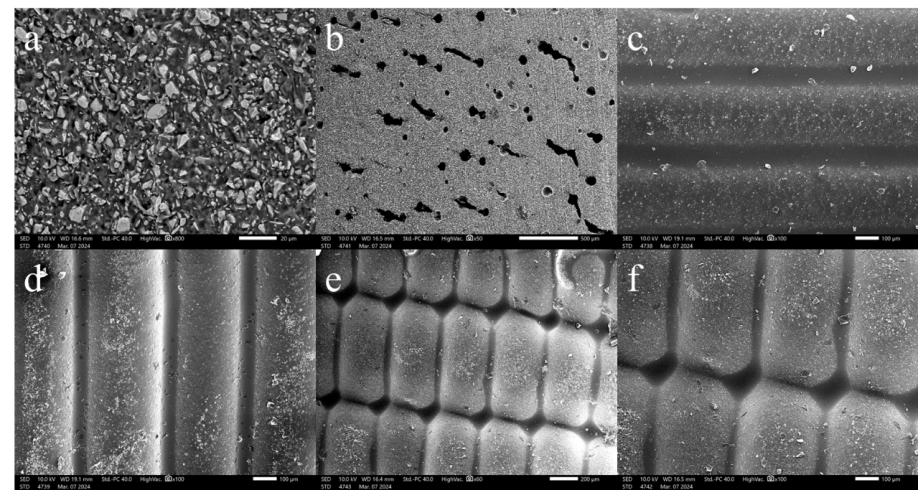


**Fig. S4** SEM images of printed samples (extrusion pressure 2.1 bar, print extrusion head 0.41 mm). (a) Characteristics of solid-phase particle distribution in the line cross-section. (b) Distribution of voids in the line cross-section of the 3D printing molding line. (c) Interline bonding interface of planar printed sample. (d) Vertical interlayer bonding state. (e-f) Print line corner detail.

**S4 Effect of NdFeB particle volume fraction on material properties**

Fig. S5 (a) shows the magnified images of the fracture surfaces of the tensile samples with NdFeB particles volume fractions of 20%, 25%, 30% and 35%, respectively. It is observed that the fracture surfaces of all the samples show horizontal fracture characteristics, which indicates that the increase of the solid phase content does not have a significant effect on the internal fracture pattern of the magnetic soft materials within the experimentally set volume fraction range.

The magnetic soft material samples with different NdFeB particle volume fractions showed significant performance differences. The bending fracture test (Fig. S5 (b)) on the 0.6 mm thick samples revealed that: when the volume fraction of NdFeB particles was 20%, the samples had excellent flexibility and did not fracture even when they were completely folded; with the increase of the volume fraction, the radius of curvature of the samples at fracture tended to increase dramatically. When the volume fraction reaches 35%, the radius of curvature at fracture increases to about 1.5 mm, and the external force required to deform the material increases significantly. This high rigidity characteristic is contrary to the design requirements of magnetic energy storage actuator for material flexibility. Considering the mechanical properties of the material and the functional requirements of the actuator, a 30% volume fraction of NdFeB particles was finally selected for the preparation of the magnetic storage actuator in this study.


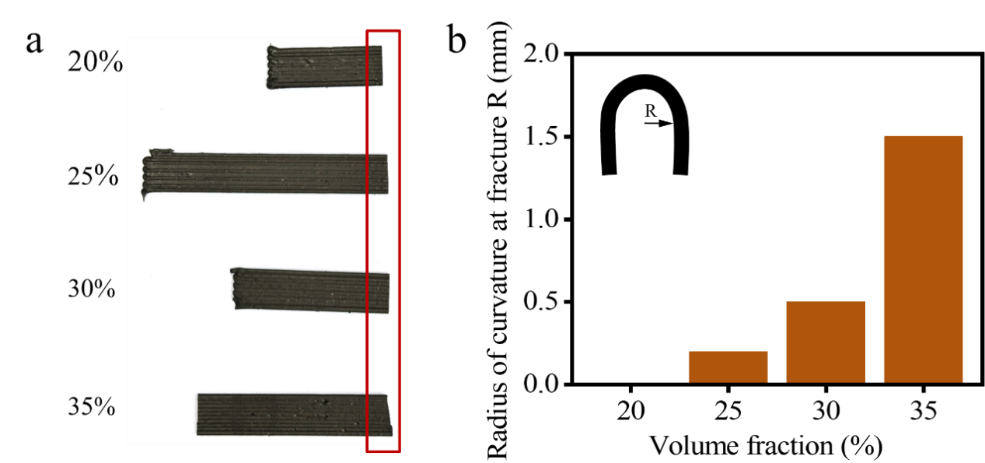


**Fig. S5** (a) Tensile fracture surface micro-morphology of samples with different NdFeB particle volume fractions (20%, 25%, 30%, 35%). (b) Comparison of the minimum radius of curvature at bending-folding fracture for samples with different NdFeB particle volume fractions.

In addition to the mechanical properties, the content of NdFeB particles also significantly affects the magnetic properties of the printing ink. Figure S6 demonstrates the magnetic hysteresis loops of materials with different NdFeB particle volume fractions (20-35 vol%). The results show that the saturation magnetization, remanent magnetization, and intrinsic coercivity of the printing inks gradually increase as the NdFeB volume fraction increases from 20 vol% to 35 vol%. This law provides an important basis for the optimisation of magnetic material printing formulations.

**Fig. S6** Effect of ndfeb particle volume fraction on magnetization hysteresis loops of printing inks

**S5 Optimization of magnetic ESUs**

In this study, the dynamics simulation under magnetic coupling is carried out for four types of units: cylindrical, cuboid, triangular prism and hollow rectangular prism. In the simulation process, one of the units is fixed and the other is allowed to move only in the Z direction under the magnetic attraction to investigate the resulting accelerated motion behavior, and the initial spacing between the two units is set to 5 mm to investigate the dynamical behavior of the units under the interaction force. Each ESU has the same bottom area and volume. In this study, we use the parametric scanning method to systematically analyze the magnetic attraction behaviors of the two units along the X-direction in Fig. S7 under the deviations of 0 mm, 1 mm, 2 mm, and 3 mm, respectively.


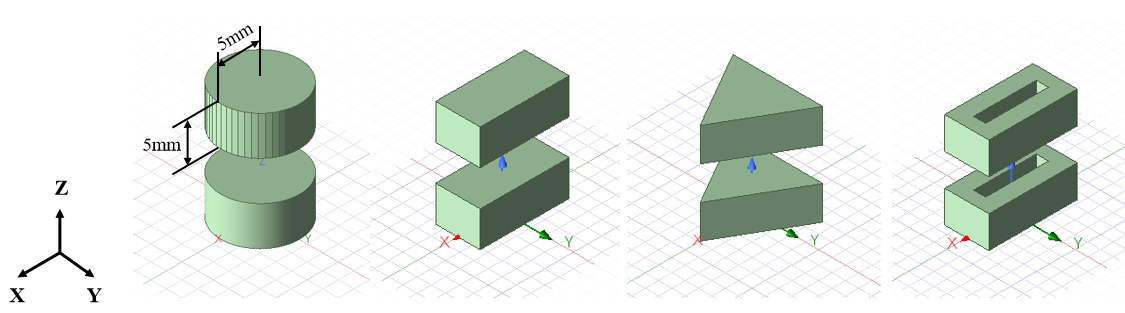


**Fig. S7** Simulation schematic of storage units attracting each other.

Fig. S8 shows the transient motion velocity response curves (in m/s) of pairs of magnetic ESUs (cylindrical, cuboid, triangular prism and hollow rectangular prism) under the action of magnetic attraction. The velocity in the vertical coordinate of the figure is the instantaneous velocity of the upper ESU accelerating along the Z direction under the magnetic attraction of the lower unit. The purpose is to quantify the difference in the dynamic performance of ESUs with different geometrical structures under the positional offset condition, and to provide a basis for the optimal selection of ESUs in the main text. The analysis results show that the four samples with height of 5 mm attract each other at a distance of 5 mm, and the maximum velocity during the motion is up to 1.6 m/s. This result confirms that by applying this type of ESU to the magnetic energy storage actuator, the actuator has the ability to accelerate the loaded object to more than 1 m/s in a very short period of time, and it provides a key basis for the design of the magnetic energy storage actuator.

Further comparison of the displacement-time curves in Fig. S8 reveals that as the offset increases, the endpoint shifts from the upper-left to the lower-right corner. This shift indicates that the intensification of the offset of the ESU leads to a significant increase in the time required to reach the target position and a significant decrease in the final velocity that can be obtained. In the initial stage, the velocity of the cylindrical unit grows most rapidly, but as the offset increases, the velocity growth rate of the cylindrical and triangular prism units decreases sharply; in contrast, the velocity of the cuboid unit decreases in a relatively gentle trend. In addition, the velocity curves of the rectangular column cells are separated from those of the other cells, which indicates that the rectangular energy storage cells can still maintain the velocity stability under larger offset conditions. It is noteworthy that the final velocity achieved by the rectangular column unit is in the leading position in all offset conditions, which is important for the application scenarios with strong demand for instantaneous energy bursts.

**Fig. S8** Transient velocity response of the ESU under lateral offset (initial pitch = 5 mm): (a) 0 mm, (b) 1 mm, (c) 2 mm, (d) 3 mm.

Fig. S9 illustrates the analytical results of the axial attraction force on the four ESUs under different displacement conditions. As shown in Fig. S9a, when the ESUs are completely aligned, the magnetic attraction force on the cylinders, cuboids, and triangular prisms are similar in magnitude, which is about 850 mN, and this phenomenon suggests that, although there are differences in the distribution of the magnetic flux density inside the different solid ESUs, the differences in the external magnetic properties of the units are small, which indicates that the geometrical shapes of the different solid units have a limited impact on the magnetic attraction or repulsion behaviors. In contrast, hollow rectangular prisms may undergo magnetically driven deformation during the magnetic attraction process due to their unique internal magnetic field distribution characteristics. Since the horizontal component of the magnetic force generated by this deformation only leads to a change in the shape of the structure and is not involved in the attraction or repulsion between the magnets, the axial attraction force on a hollow rectangular prism is relatively small under the same displacement conditions.

The magnetic attraction forces exerted on the units show a significant trend as the offset increases. The magnetic attraction forces on triangular prisms and cylinders decreased continuously with the increase of the offset. When the offset reaches 2 mm (Fig. S9c), the maximum magnetic attraction force of the triangular prism decreases from the initial 856 mN to 564 mN; when the offset increases to 3 mm (Fig. S9d), the maximum magnetic attraction force of the cylinder decreases to 499 mN, and that of the triangular prism is even lower than 400 mN, which is lower than that of the hollow rectangular prism. In contrast, the rectangular column and the hollow rectangular prism show high stability. Even at an offset of 3 mm, the maximum magnetic attraction force of the rectangular column is still 619 mN, which is about 1.5 times that of the triangular prism, and the magnetic attraction force of the hollow rectangular prism reaches 566 mN, which is not significantly different from that of the rectangular column.

**Fig. S9** Axial attractive-displacement responses of four ESUs at an initial spacing of 5 mm: comparison of (a) 0 mm, (b) 1 mm, (c) 2 mm, and (d) 3 mm offset.

Moment characteristics are the key indexes to measure whether the ESU can realize accurate repositioning during operation. Here, Fig. S10 (a)~(c) systematically shows the variation rule of the moment under different displacement states of the ESU under the working condition of 1~3 mm lateral offset.

The analysis results show that the moments of the cylinder and triangular prism are relatively low at an offset of 1 mm (Fig. S10a), which is only about 500 μN·m. The moments of the cylinder and triangular prism do not show a significant increase with the further increase of the offset, and their peaks are always maintained at about 600 μN·m. In contrast, the rectangular columns show very different moment characteristics. At 1 mm offset, the maximum moment of the hollow rectangular prism reaches 1.008 μN·m; when the offset is increased to 2 mm (Fig. S10b), the maximum moment increases to 1.532 mN-m; and at 3 mm offset (Fig. S10c), the maximum moment further climbs to 1.843 mN-m. The maximum moment of the rectangular column is 1.008 mN·m at 1 mm offset (Fig. S10b), which is the same as that of the rectangular column.

The higher moment value means that the rectangular ESU is able to utilize the moment more effectively to achieve precise alignment adsorption during actual operation, thus significantly reducing the energy loss due to adsorption eccentricity. On the contrary, the triangular prism and cylindrical ESUs, due to their lower moment levels, indicate that they have limited ability to eliminate the eccentricity error, and are more prone to energy loss in practical applications, which affects the overall performance of the system.

**Fig. S10** Effect of lateral offset on the moment response of the ESU (initial spacing = 5 mm): (a) 1 mm, (b) 2 mm, (c) 3 mm.

**S6 Magnetic Ejection Actuator**

A printed prototype of the magnetic energy storage ejection actuator is shown in Figure S10.

**Fig. S11** Structure of the stored magnetic energy ejection actuator, scale bar: 5mm. Structural dimensions are in mm.

When the stored magnetic energy actuator is in operation, its running attitude depends on the mutual attraction of the two magnetic ESUs. In order to accurately analyze the magnetic field distribution characteristics under this working condition, the COMSOL Multiphysics simulation software is used in this study to carry out the simulation analysis of magnetic field distribution. As shown in Fig. S12, the magnetic flux density distributions at different cross-sections are significantly different due to the design of the actuator's shaped structure. This figure selects and shows the magnetic flux density distribution characteristics of two key cross sections:

At section Ⅰ, the ESUs on both sides are relatively close to each other due to the lack of notch structure. Analyzing the magnetic induction distribution, it can be seen that the peak magnetic flux density is mainly concentrated in the corner region, while the magnetic flux density in the plane region is relatively low, and the overall maximum magnetic flux density can be up to 0.205 T. It is worth noting that in this attitude, the distribution of magnetic inductance under the ESUs on both sides presents a high density of magnetic inductance, which suggests that the attraction force generated in this region will be the main source of the power for the initial motion of driving the actuator.

Cross-section Ⅱ demonstrates the cross-section flux density distribution in the center plane of the entire magnetic energy storage actuator, and the color layout is shown to be adopted as the same as that of cross-section Ⅰ, and it can be clearly seen that the flux density at cross-section Ⅱ is weaker than that at cross-section Ⅰ, due to the lack of structure and the longer distance. Further observation of the magnetic inductance closure paths reveals that the percentage of magnetic inductance closure within a single ESU is higher at section Ⅱ, while the magnetic inductance connections across units are relatively few. This phenomenon suggests that the part of the actuator containing the notch structure has a more limited contribution to the overall magnetic attraction driving force at this operating attitude. Figure S12 illustrates the ejection process of a hot weather responsive stored magnetic energy ejection actuator on a seed sub.

The seed motion was tracked by recording the seed ejection trajectory and using Tracker software. The velocity of the seeds was obtained by displacement-time curve fitting, and their kinetic energy (Ek) was calculated by the formula：$E_{k}={mv^{2}}/2$. For example, if the mass of 7mm seed is ***m*** (0.181 g) and the measured ejection speed is v (1.119 m/s), then its final kinetic energy Ek = 1/2 mv2 = 0.113 mJ.

The maximum magnetic energy density E_m_ ≈ 0.5 mJ is estimated by COMSOL simulation of the flux density distribution; and its efficiency percentage: $\delta={E_{k}}/{E_{m}\times100\%\approx22.6\%}$. It should be emphasised that this value is an ideal model estimation, and the actual value will be low due to losses.


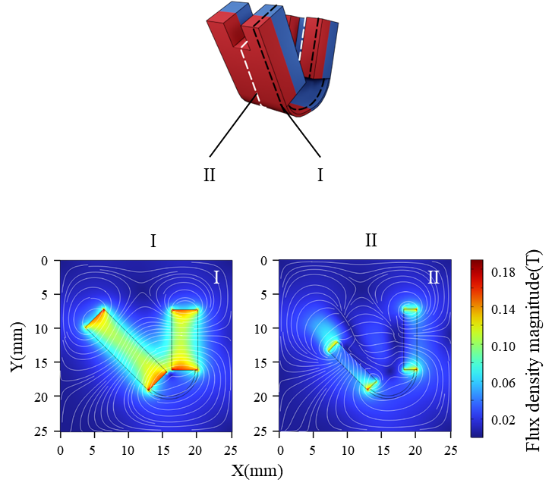


**Fig. S12** Simulation results of magnetic flux density distribution in double cross section of stored magnetic energy ejection actuator (opening angle θ=45°).


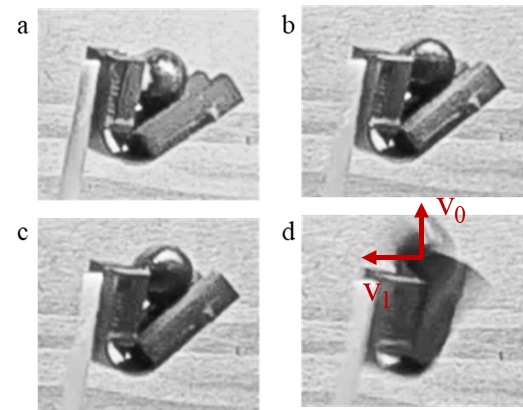


**Fig. S13** Ejection process of seeds by a stored magnetic energy ejection actuator in response to hot weather.

**S7 Bionic Gripping Actuator**

The structure of the magnetic energy storage gripping actuator is shown Fig. S14. The right side uses a hollow rectangular prism unit as the ESU, and its internal gap helps in unit fixation and external signal conduction. The middle ESU is mainly responsible for the final magnetic suction. The overall design of the actuator is thin and long, which can adapt to the suction deviation caused by different gripping positions. The whole consists of a rectangular energy storage body and two outwardly extending connection piece for connecting to the hollow rectangular prism ESU. It was found that designing the connection piece as a protruding solid stepped structure would change the inter-unit repulsion characteristics, while the optimized connection piece ensured fast fixation and release of the ESU while minimizing the impact on the magnetic properties.

**Fig. S14** Structure of the stored magnetic energy bionic gripping actuator, scale bar: 10mm. Structural dimensions are in mm.

The epimeter magnetic analyzer was used to analyze the surface magnetic field of the gripping actuator, and the results are shown in Fig. S15. The results are shown in Fig. S15. The analysis shows that the magnetic field distribution is in accordance with the design expectation, and the flux density extremes are located at the three ESUs, and the magnetic pole direction change occurs in the middle of the actuator. Specifically, the flux density at the left ESU reaches the maximum value of about 518 Gs; the longer ESU in the middle has a relatively low flux density of 156 Gs due to its thin thickness; and the right hollow rectangular prism ESU generates a weaker external magnetic field than the left unit due to its thickness being smaller than that of the left unit and the existence of an internal gap. Further analysis of the magnetic field distribution of the hollow rectangular prism reveals that it shows the characteristics of high on both sides and low in the middle, which is attributed to the hollow structure in the center region and the aggregation effect of the magnetic field in the corners, resulting in a lower flux density in the middle part.

**Fig. S15** Surface magnetic flux density distribution of the stored magnetic energy gripping actuator.

In this study, a series of grasping actuators were prepared by varying the height h of the hollow rectangular prism ESU (with the value range of 1-4 mm), and their motion attitude after ejection was systematically analyzed, and the results are shown in Fig. S16. Among them, Fig. S16 (a) and Fig. S16 (b) show the changes of X and Y coordinates of the endpoints of the hollow rectangular prism ESU with different heights ***h*** within 0.12s, using the initial position of the ejected unit as the coordinate origin. According to the structural design principle, the initial displacement of the endpoint in the X-direction is mainly driven by the magnetic energy ejection, while the initial motion in the Y-direction is mainly driven by gravity and the binding force of the ESU underneath.

The first half of Fig. S16 (a) shows that the X-coordinate motion trends of the different actuators are generally similar, but the displacement of the endpoints in the X-direction increases with increasing ***h*** and eventually stabilizes, indicating that there is a saturating effect of increasing the thickness of the storage units on both sides on the motion gain of the ejection unit. In the second half of Fig. S16 (a), the difference between the curves is significant, indicating that the lower binding force starts to affect the X-direction motion of the endpoints, with the 4-mm-height ESU being the least affected. In Fig. S16 (b), the curves have a high degree of overlap at the initial stage, reflecting the consistency of the gravitational effect, while the time for the endpoints to reach the Y-direction nadir is gradually delayed with the increase of ***h***, which is attributed to the decrease in the rate of change of the velocity for the same constraint force due to the increase of gravity.

Fig. S16 (c) visualizes the magnitude and direction of the velocity of the endpoints through arrows. The results show that the actuator endpoint with h = 1 mm has the maximum integrated travel velocity; while the maximum range of motion does not correspond to the actuator with ***h = 4 mm***, but occurs at ***h = 3.25 mm***. This result indicates that as the mass of the ESU increases, its magnetic energy increment is not sufficient to offset the negative effect of the weight increase on the kinematic performance.


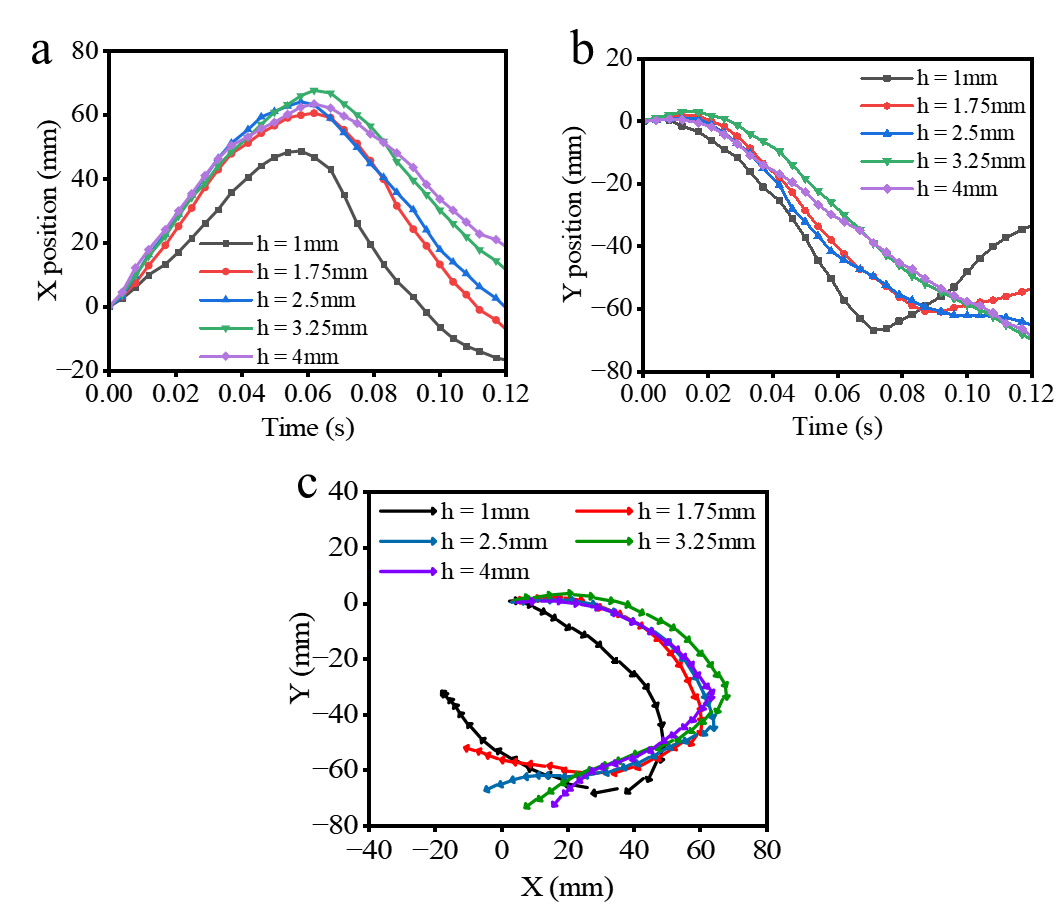


**Fig. S16** Comparison of the effect of the stored magnetic energy gripping actuator parameter ***h*** on the dynamic behavior of the ejector unit endpoint. (a) X-axial displacement-time curves of the endpoint for different values of ***h***. (b) Y-axial displacement-time response comparison. (c) Time-varying characteristics of the velocity direction vector of the endpoint motion.

**S8 Logic Gated Jump Actuator**

The process of DIW printing of the locking material is shown in Fig. S17 (a). First, the calcium carbide particles were crushed and milled to obtain a powder suitable for DIW; subsequently, the milled calcium carbide powder was dispersed in PVP ethanol solution, and a uniform ink was formed after thorough mixing; finally, a DIW printer was used to form the lines. The resulting lines are shown in Fig. S17 (b). By adjusting the specification of the extrusion head, the diameter of the line can be precisely controlled, thus realizing the effective control of the water response time of the actuator. The molded line was immersed in molten phase change wax at 45℃, and logic response "locking material" was prepared after the wax completely coated the line.


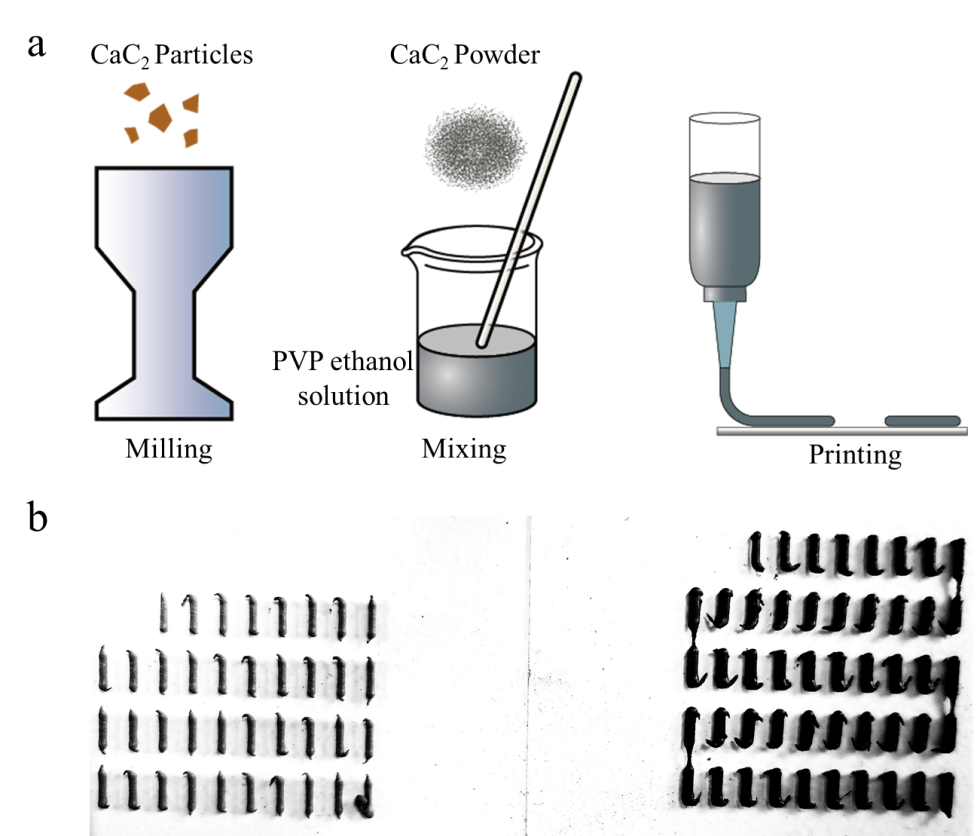


**Fig. S17** Fabrication process of locking structure based on ink DIW technology and its performance regulation mechanism. (a) Schematic of the DIW printing process for the locking structure. (b) Comparison of the effects of printing parameters on functional properties (extrusion head diameter parameter comparison: left 1.0 mm; right 1.4 mm), demonstrating the tunable characteristics of the actuator's environmental response rate.

A 'locking' structure rigidly secures the logic-responsive jump actuator, which has a groove at the center of its right end (Fig. S18). By shortening the distance between the two ends of the actuator, the design effectively enhances the repulsion between the ESUs, so that the accumulation of magnetic energy in the fixed state of the structure reaches a near-maximum value. Under these conditions, once the rapid failure mechanism of the "locking material" is triggered, an instantaneous release of magnetic energy can be realized. Therefore, the logic response behavior of the entire bouncing actuator can be directly controlled by regulating the logic response characteristics of the "locking material".

**Fig. S1****8** Structure of a logic gated jump actuator. Scale bar: 5mm. Structural dimensions are in mm.

Fig.S19 presents the surface magnetic field distribution data of the logic-responsive actuator. The analysis results show that the peak of the actuator magnetic flux density is located in the area of the energy storage unit (ESU), which can reach up to 468 Gs. In contrast, the magnetic flux density at the connection area of the two ends of the artificial design is significantly lower, and the magnetic flux density in the notch area is even lower. This phenomenon suggests that the strength of the magnetic field at the connection area has less influence on the overall bouncing kinematic performance of the actuator.

**Fig. S19** Distribution of surface magnetic flux density for a logic-responsive magnetic energy storage jump actuator.

Fig. S20 shows the jumping dynamic sequence captured by the high-speed camera when the actuator is placed diagonally. The analysis shows that the jumping motion of the actuator is mainly driven by the support at the lower connection part. During the jump preparation phase, the lower side ESU is the first to deform and its connecting end quickly forms a support contact with the ground. Under the action of this support force, the lower ESU flips to a state nearly perpendicular to the ground at a high speed, and at the same time triggers the rapid release of magnetic energy, pushing the upper ESU upward. Thanks to the kinetic energy of the upper ESU and the reaction force of the ground, the actuator obtains an initial velocity diagonally forward, realizing a jump and a quick roll in the air. This unique motion pattern not only enables the actuator to perform long-distance diagonal jumps, but also to spread spores and other materials over a large area through the tumbling motion.


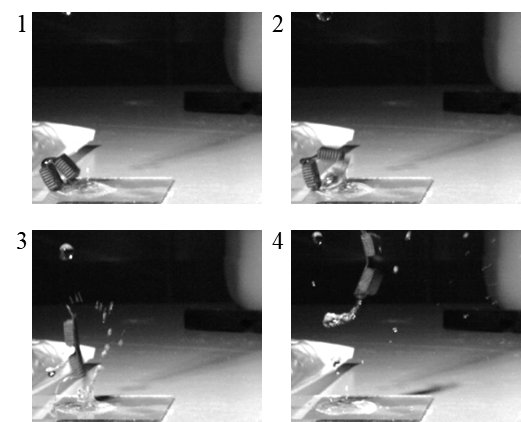


**Fig. S20** Jumping attitude of the actuator in oblique placement.

In order to investigate the influence of the placement attitude and angle on the jumping performance of the actuator, we selected the actuators with different placement attitudes for the experiment, and their jumping performance curves are shown in Fig. S21. By comparing the motion state of the actuator's centre of mass, we can quantitatively analyse the effect of different placement attitudes on the jumping performance.

As can be seen from Fig. S21a, with the increase θ, the jump distance of each actuator tends to increase, but the jump height decreases gradually; among them, the inclined placement actuator is much higher than the other two types of placement in terms of jump height and distance. Observing Fig. S21b, it can be seen that although the speed in x-direction fluctuates due to the rotation of the actuator itself, the overall speed is stable in a specific interval and does not decrease significantly; and the speed in x-direction of the inclined placement actuator is significantly better than that of the other two cases, which makes it able to achieve a longer jumping distance within the same period of time.

From Fig. S21c, it can be seen that all the actuators have the same pattern of velocity change in the y-direction: first a sharp increase, then a nearly linear slow decrease under the effect of gravity, which is in line with the characteristic of the effect of gravity on the velocity of motion. Although the maximum velocities in the y-direction obtained by each actuator are relatively similar, the actuator with Ⅲ/θ=0° is able to maintain the high velocity for a longer period of time, and thus obtains a larger jump height. This result suggests that inclined placement achieves a higher energy conversion rate than other placement methods where magnetic energy is wasted due to actuator rotation.


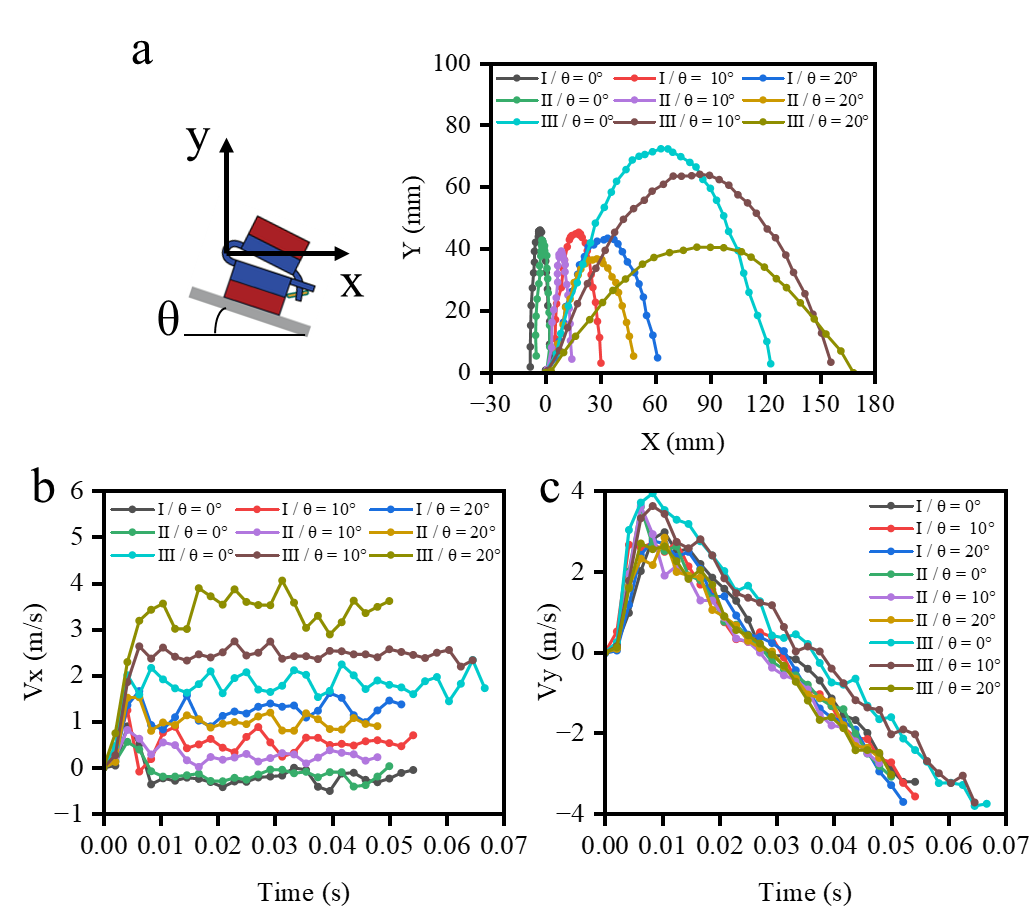


**Fig. S21** Comparison of the jump performance of the actuators in different placement attitudes. (a) Motion path of the jumping actuator centre of mass. (b) Variation of the velocity of the centre of mass in the x-direction with time. (c) Variation of the velocity of the centre of mass in the y-direction with time.

We compare the jumping performance of the obliquely placed actuator with the existing related studies, and the results are shown in Fig. S22. It can be clearly seen from the figure that the actuator proposed in this study significantly outperforms the existing actuators reported in the literature in terms of jumping distance (120-180 mm) and jumping height (40-75 mm), and achieves a balance between “long jump” and “high jump”, with better overall performance.

**Fig. S22** Comparison of actuator jump performance (jump height and jump distance) with other reported soft actuators^[1-10]^.

**References**

[1] Kim Y, Van Den Berg J, Crosby A J. Autonomous snapping and jumping polymer gels[J]. Nature Materials, 2021, 20(12): 1695-1701.

[2] Wu Y, Yim J K, Liang J, et al. Insect-scale fast moving and ultrarobust soft robot[J]. Science Robotics, 2019, 4(32):eaax1594.

[3] Arazoe H, Miyajima D, Akaike K, et al. An autonomous actuator driven by fluctuations in ambient humidity[J]. Nature Materials, 2016, 15(10): 1084-1089.

[4] Duduta M, Berlinger F C J, Nagpal R, et al. Electrically-latched compliant jumping mechanism based on a dielectric elastomer actuator[J]. Smart Materials and Structures, 2019, 28(9): 09LT01.

[5] Hu Y, Liu J, Chang L, et al. Electrically and Sunlight‐Driven Actuator with Versatile Biomimetic Motions Based on Rolled Carbon Nanotube Bilayer Composite[J]. Advanced Functional Materials, 2017, 27(44):1704388.

[6] Yu K, Ji X, Yuan T, et al. Robust Jumping Actuator with a Shrimp‐Shell Architecture[J]. Advanced Materials, 2021, 33(44):2104558.

[7] Huang X, Kumar K, Jawed M K, et al. Chasing biomimetic locomotion speeds: Creating untethered soft robots with shape memory alloy actuators[J]. Science Robotics, 2018, 3(25): eaau7557.

[8] Ahn C, Liang X, Cai S. Bioinspired Design of Light‐Powered Crawling, Squeezing, and Jumping Untethered Soft Robot[J]. Advanced Materials Technologies, 2019, 4(7): 1900185.

[9] Chen R, Yuan Z, Guo J, et al. Legless soft robots capable of rapid, continuous, and steered jumping[J]. Nature Communications, 2021, 12(1): 7028.

[10] Xu L, Xue F, Zheng H, et al. An insect larvae inspired MXene-based jumping actuator with controllable motion powered by light[J]. Nano Energy, 2022, 103: 107848.

1. * **Corresponding author:** Lu Zhang; Xueli Zhou

   **E-mail:** [lu_zhang@jlu.edu.cn;](mailto:lu_zhang@jlu.edu.cn;) xlzhou@jlu.edu.cn [↑](#footnote-ref-0)
